# Diverged composition and regulation of the *Trypanosoma brucei* origin recognition complex that mediates DNA replication initiation

**DOI:** 10.1093/nar/gkw147

**Published:** 2016-03-06

**Authors:** Catarina A. Marques, Calvin Tiengwe, Leandro Lemgruber, Jeziel D. Damasceno, Alan Scott, Daniel Paape, Lucio Marcello, Richard McCulloch

**Affiliations:** The Wellcome Trust Centre for Molecular Parasitology, Institute of Infection, Immunity and Inflammation, University of Glasgow, Sir Graeme Davis Building, 120 University Place, Glasgow, G12 8TA, UK

## Abstract

Initiation of DNA replication depends upon recognition of genomic sites, termed origins, by AAA+ ATPases. In prokaryotes a single factor binds each origin, whereas in eukaryotes this role is played by a six-protein origin recognition complex (ORC). Why eukaryotes evolved a multisubunit initiator, and the roles of each component, remains unclear. In *Trypanosoma brucei*, an ancient unicellular eukaryote, only one ORC-related initiator, TbORC1/CDC6, has been identified by sequence homology. Here we show that three TbORC1/CDC6-interacting factors also act in *T. brucei* nuclear DNA replication and demonstrate that TbORC1/CDC6 interacts in a high molecular complex in which a diverged Orc4 homologue and one replicative helicase subunit can also be found. Analysing the subcellular localization of four TbORC1/CDC6-interacting factors during the cell cycle reveals that one factor, TbORC1B, is not a static constituent of ORC but displays S-phase restricted nuclear localization and expression, suggesting it positively regulates replication. This work shows that ORC architecture and regulation are diverged features of DNA replication initiation in *T. brucei*, providing new insight into this key stage of eukaryotic genome copying.

## INTRODUCTION

Genome transmission is central to life and requires replication to generate new genome copies, which are then passed to progeny. In all cellular organisms DNA replication is initiated at genomic sites termed origins, which are bound by initiator factors. Binding of initiators allows recruitment and activation of the replicative helicase, which leads to opening of the origin DNA, recruitment of the remainder of the replication machinery and initiation of DNA synthesis (1–3). Though all initiators appear related, in that they belong to the AAA+ superfamily of NTPases (4,5), their functional architecture differs between prokaryotes and eukaryotes. In prokaryotes an origin is bound by a single initiator protein, which in bacteria oligomerises during recruitment of the replicative helicase. In contrast, and for reasons that remain unclear, the origin-binding initiator has evolved into a multiprotein complex in eukaryotes.

Eukaryotic genomes contain many origins of replication, each recognized by a conserved six-subunit origin recognition complex (ORC) (6). Like prokaryotic initiator factors (DnaA in bacteria and Orc1/Cdc6 in archea) (7,8), five of the six subunits of eukaryotic ORC (Orcs1–5, but not Orc6) belong to the DnaA/CDC6/ORC clade of the AAA+ superfamily of ATPases (9). Once ORC is bound to an origin, Cdc6 (Cell Division Cycle 6), another AAA+ ATPase and a paralogue of Orc1 (6,10), is recruited. Cdc6 binding induces conformational changes in ORC (11) that stabilize ORC-origin interaction (12) and allow recruitment, mediated by Cdt1 (13–15), of the heterohexameric MCM2–7 replicative helicase (16). All six minichromosome maintenance (MCM) subunits are also AAA+ ATPases (17), and the structure formed by MCM2–7-Cdt1-Cdc6-ORC interaction is termed the pre-replication complex (pre-RC). Formation of the pre-RC at origins takes place from mitosis to late G1 phase of the cell cycle and is known as origin licensing (1,2).

ORC was first purified from *Saccharomyces cerevisiae* (18). Subsequently, ORCs were identified in *Xenopus laevis* (19), *Drosophila melanogaster* (20) and *Schizosaccharomyces pombe* (21), and characterization of the subunits revealed their conservation with *S. cerevisiae* and in many other eukaryotes, including mammals (6). Orcs1–5 and Cdc6 share a common structure: each contains a central or N-terminal AAA^+^ ATPase domain and, downstream, a winged helix (WH) domain that, together, mediate DNA binding (22–24). Most eukaryotic Orc1 subunits possess N-terminal homology with Sir3, including a bromo-adjacent homology (BAH) domain (25), which underlies Orc1's role in transcriptional silencing (26,27). Orc6 appears to be unrelated to the other ORC subunits (28), lacking discernible AAA+ homology (6), though structural studies have revealed homology between the N-terminus of metazoan Orc6 and the transcription factor TFIIB (29), which may contribute to DNA binding (30). Structural analysis by electron microscopy (EM) and, recently, by crystallography has revealed the order of Orc subunit interaction within ORC, as well as how Cdc6 directs recruitment of Cdt1-MCM2–7 in the pre-RC (11,22,31–33). The broadly conserved EM-derived structures of ORC from *S. cerevisiae* (34) and *D. melanogaster* (35) are consistent with interlocking of the AAA+ and WH domains of the Orc subunits being central to the function of the complex (22). Such interlocking is likely to be the basis for the conformational changes associated with ORC assembly and DNA interaction, which are due to ATP binding and hydrolysis by the Orc subunits (22,33,36). Indeed, these ATP-driven conformational changes extend beyond ORC, with the ATPase activity of Cdc6 further changing ORC structure and modulating interaction with the other pre-RC components (11,12,32,37,38). Despite this, it remains unclear why six AAA+ ATPases factors are needed for ORC-Cdc6 function, and what function each ORC subunit provides. In archaea the MCM replicative helicase is also hexameric (39) but is recruited to an origin by a single protein, termed Orc1/Cdc6, which is related to both eukaryotic Orc1 and Cdc6 and fulfils the functions of the two proteins (40). Archaeal Orc1/Cdc6 proteins use ATPase activity and co-operative interactions between monomers to distort the origin DNA (23,24,41), suggesting broad functional similarity to eukaryotic ORC-Cdc6. Why there is such an apparent gulf between the architectures of archaeal and eukaryotic initiators is unclear, in particular because growing evidence suggests that eukaryotes arose from an archaeal ancestor(42).

To date, ORC architecture has only been explored in the opisthokont supergroup of eukaryotes, which includes yeast, *D. melanogaster* and mammals. Relatively little work has examined DNA replication in protists, a vast grouping of unicellular eukaryotic microbes that provides most of the diversity in the eukaryotic domain (43–45). In *Tetrahymena thermophila*, a multisubunit ORC complex has been described, some of whose components are related to the canonical eukaryotic subunits, but the overall architecture remains unresolved (46,47). Homology searches reveal that it is surprisingly difficult to identify homologues of all six Orc subunits, or Cdc6, in many eukaryotic organisms belonging to the non-opisthokont supergroups (48,49). Whether this is because of lineage-specific gene losses from an ancestral six subunit ORC-Cdc6 organization (50), or if the opisthokont initiation machinery may not be as universal in eukaryotes as anticipated, is currently unclear.

The kinetoplastida is a well-studied order of eukaryotic microbes that belongs to the excavata supergroup (51) and contains a number of important human and animal parasites, such as *Trypanosoma brucei*. Genome sequencing of *T. brucei* and related kinetoplastid parasites identified only a single ORC-related protein (52), which contains well-conserved AAA+ ATPase motifs and some evidence of a C-terminal WH domain (53), but lacks N-terminal sequences found in other eukaryotic Orc1 subunits, including the BAH domain. The structural similarity of this protein to Orc1/Cdc6 in archaea has led to adoption of the name ORC1/CDC6, an analogy that may be supported functionally by the ability of *T. brucei* ORC1/CDC6 (TbORC1/CDC6) to complement *S. cerevisiae cdc6* temperature sensitive mutants (53). TbORC1/CDC6 has been shown to act in *T. brucei* nuclear DNA replication, both through impairment of nucleotide analogue incorporation after RNA interference (RNAi) (54) and localization of the protein at mapped replication origins in the genome (55). A number of TbORC1/CDC6-interacting factors have subsequently been identified, raising the possibility that an ORC is present. However, many of the TbORC1/CDC6 interactors are highly diverged in sequence from canonical ORC subunits (49) and none has been shown to have a role in replication. One such factor has been named TbORC1B, based on its identification by weak homology with Orc1 and the presence of AAA+ ATPase motifs (56). Amongst three further TbORC1/CDC6 interactors (49), one (named TbORC4) appears to be a distant orthologue of Orc4, while the two others (Tb3120 and Tb7980), though displaying weak evidence for ATPase motifs, cannot be assigned ORC subunit orthology because primary sequence-based homology searches reveal only kinetoplastid homologues (49). Here, we show that TbORC1B, TbORC4 and Tb3120 all act in nuclear DNA replication, and provide evidence that TbORC1/CDC6 and TbORC4 are present in a high molecular complex in the parasite, suggesting the presence of a diverged ORC. Within this complex we can also detect the helicase subunit TbMCM3, suggesting the presence of a stable pre-RC-like complex. By examining the subcellular localization of all currently identified TbORC1/CDC6 interacting factors, we show that TbORC1B is not a static component of the *T. brucei* ORC and instead shows S-phase limited expression, suggesting that this Orc1-like factor adopts an unorthodox, positive regulatory role in *T. brucei* nuclear DNA replication.

## MATERIALS AND METHODS

### *Trypanosoma brucei* strains, growth and transformation

*Trypanosoma brucei* procyclic form (PCF) cells, strain Lister 427 pLew29-pLew13 (57), were used for RNAi assays, while PCF TREU 927 cells were used for endogenous epitope tagging, microscopy, immunoprecipitation and gel filtration assays. TREU927 cells expressing TbORC1/CDC6 endogenously tagged with 12myc at the C-terminus and with the remaining TbORC1/CDC6 allele deleted, as well as expressing TbMCM3 endogenously tagged with 6HA, have been described previously (49). PCF cells were cultured in SDM-79 (Gibco) supplemented with 10% (v/v) heat-inactivated fetal bovine serum (FBS, Sigma-Aldrich), 1% (v/v) penicillin-streptomycin solution (Gibco), and 5 μg.ml^−1^ of haemin (Sigma-Aldrich), at 27°C, in non-vented flasks. Whenever required, the medium was supplemented with the appropriate selective drugs as follows: 50 μg.ml^−1^ of hygromycin; 10 μg.ml^−1^ of G418, zeocin or blasticidin; 1 μg.ml^−1^ of puromycin (all InvivoGen). Bloodstream form (BSF) Lister 427 cells were used for endogenous tagging and subcellular localization by microscopy, and cultured in HMI-9 (Gibco) supplemented with 20% (v/v) FBS (Sigma-Aldrich) and 1% (v/v) of penicillin-streptomycin solution (Gibco). For RNAi, BSF Lister 427 cells, strain 2T1 (58), were used. BSF cells were routinely maintained in HMI-11 medium, composed of HMI-9 medium supplemented with 10% (v/v) FBS (Sigma-Aldrich), and 1% (v/v) of penicillin-streptomycin solution (Gibco); when assaying EdU uptake, however, the cells were cultured in HMI-11 thymidine-free medium consisting of Iscove's Modified Dulbecco's Medium (IMDM) (Gibco), 10% (v/v) FBS (Gibco, tetracycline free), 1% (v/v) penicillin-streptomycin solution (Gibco), 4% (v/v) HMI-9 mix (0.05 mM of bathocuproine disulphonic acid, 1 mM of sodium pyruvate and 1.5 mM of L-cysteine) (Sigma Aldrich), 1 mM hypoxanthine (Sigma Aldrich) and 0.0014% 2-mercaptoethanol (Sigma Aldrich). In all cases, BSF cells were incubated at 37°C and 5% CO_2_ in vented flasks. Whenever required, the medium was supplemented with the appropriate selective drugs as follows: 5 μg.ml^−1^ of hygromycin; 2.5 μg.ml^−1^ of G418 or phleomycin; 10 μg.ml^−1^ of blasticidin; 0.2 μg.ml^−1^ of puromycin (all InvivoGen). Both PCF cells and BSF cells were transformed as described elsewhere (49).

### Endogenous epitope tagging

TbORC1B, TbORC4 and Tb3120 were endogenously tagged, at the C-terminus, with 12 copies of c-myc (12myc) using constructs derived from the pNAT^12M^ plasmid (58), as described previously (49). The 3′ end of the ORF of the gene of interest, with the exclusion of the stop codon, was PCR-amplified using the following primers: TbORC1B, GCATAAGCTTACAACGAGACAGTCAAATCG and GCATTCTAGACAGGGATAAAATGCCCTTGA; TbORC4, CCCAAGCTTCGTTTCTGCTGTCTTTGGGG and CCCTCTAGACACGAGGCTGCGTAATC; Tb3120, CCCAAGCTTAGTGCATGGTATAGACGAA and CCCTCTAGATGCCTCCACTGGAGCTCCAC. In each case, the forward primer contained a HindIII restriction site, while the reverse contained an XbaI site, allowing cloning of each gene fragment into pNAT^12M^. The resulting plasmids were linearized prior to transfection into either PCF or BSF cells, using the following restriction enzymes: ClaI (TbORC1B), SmaI (TbORC4) or NsiI (Tb3120). For tagging of TbORC1/CDC6 the construct generated in (49) was used. Transformant PCF cells were selected with 15 μg.ml^−1^ blasticidin, while BSF cells were selected with 10 μg.ml^−1^ blasticidin. Tb7980 was endogenously tagged with 12myc at the N-terminus using a construct modified from the pEnT6B plasmid (59). In this case, two fragments were PCR-amplified: the 5′ end of the Tb7980 ORF (excluding the start codon), using the primers GCATACTAGTGCAGCCCAAACACCACGCA (containing the SpeI restriction site) and GCATGGTACCCACGACGAAGTGAAGCTCA (containing the NotI site); and a section from the 5′ intergenic region upstream of the Tb7980 ORF, using the primers GCATGGTACCGACATGCCGTGACGAACTC (containing the KpnI site) and GCATGGATCCACGACGGGGAAACAGAACG (containing the BamHI site). The resulting plasmid was then linearized (NotI) prior to transfection into either PCF or BSF cells. Transformant cells were selected with 15 μg.ml^−1^ blasticidin (PCFs) or 10 μg.ml^−1^ blasticidin (BSFs).

### RNA interference analysis in PCF cells

For RNAi analysis in PCF cells, a modified version of the stem-loop pLew111 construct (60) containing the *BLE* resistance marker, and a fragment of the human *PLK1* gene within the HindIII/BamHI linker was used. This construct encloses two cloning sites, HindIII/XhoI and AflII/BamHI, which allow the sequential insertion, in a head-to-head configuration, of two identical PCR products, each flanked with one of the two different restriction site combinations. The region of each gene to be PCR-amplified (sizes between 400 and 600 bp), as well as the best pair of primers to use, was chosen using RNAit (http://trypanofan.bioc.cam.ac.uk/software/RNAit.html). For each gene, two identical fragments were PCR-amplified using the two sets of primers, so that one PCR fragment (inserted into the plasmid in the sense orientation) was flanked by the HindIII and XhoI restriction sites, while the other (inserted into the plasmid in the anti-sense orientation) was flanked by the BamHI and AflII sites. The following primers were used: TbORC1/CDC6—CCCCAAGCTTGAAGCCCACAGCTGTCTTTC, CCCCCTCGAGTTCTCCGGCAACTTGTAACC, CCCCGGATCCGAAGCCCACAGCTGTCTTTC and CCCCCTTAAGTTCTCCGGCAACTTGTAACC; TbORC4—CCCCAAGCTTCACGTTGTATCCCCTTGCTT, CCCCCTCGAGTTCAGTTTCGGCGAAGTTCT, CCCCGGATCCCACGTTGTATCCCCTTGCTT and CCCCCTTAAGTTCAGTTTCGGCGAAGTTCT; TbORC1B— CCCCAAGCTTCTATCGGCTGAGTACGCCTC, CCCCCTCGAGTTTGCGATTTGACTGTCTCG, CCCCGGATCCCTATCGGCTGAGTACGCCTC and CCCCCTTAAGTTTGCGATTTGACTGTCTCG; Tb3120—CCCCAAGCTTCTAACGGCTCAGTTTCTCGG, CCCCCTCGAGTTGGCAAAAGATTCCTCACC, CCCCGGATCCCTAACGGCTCAGTTTCTCGG and CCCCCTTAAGTTGGCAAAAGATTCCTCACC. The two PCR products were sequentially cloned into the vector, and the resulting constructs were subsequently linearised with NotI and transfected into PCF 29–13 cells. Transformant cells were selected with 10 μg.ml^−1^ zeocin, 50 μg.ml^−1^ hygromycin and 10 μg.ml^−1^ neomycin. For RNAi analysis, each cell line was diluted into two different cultures of 5.5 × 10^5^ cells. ml^−1^. To one of these, 2 μg.ml^−1^ tetracyclin (Calbiochem®, Merck Millipore) was added (Tet + sample) to induce the gene-specific RNAi, while the other culture was used as the non-induced (Tet-) sample. The cell density in both cultures was then assessed post-tetracycline addition using a Neubauer improved hemocytometer. At 6, 12 and then every 24 h, ∼10^7^ cells were collected for EdU incorporation and cell cycle (DAPI staining of nuclear and kinetoplastid DNA) analysis (described in specific sections). In parallel, ∼10^7^ cells were collected for cell cycle analysis by flow cytometry. Briefly, cells were centrifuged at 1620 g for 10 min, washed once in 1x PBS supplemented with 5 mM EDTA, and re-suspended in 300 μl of 1x PBS supplemented with 5 mM EDTA, to which 700 μl of Methanol (cooled at 4°C) was added in a drop-wise fashion while vortexing (final fixing solution of 70% (v/v) Methanol), and left at least overnight at 4°C. The cells were then centrifuged at 1620 g for 10 min at 4°C, washed once in 1x PBS supplemented with 5 mM EDTA, and finally re-suspended in 1 ml of 1x PBS supplemented with 5 mM EDTA, 10 μg.ml^−1^ propidium iodide (PI; Sigma-Aldrich), and 10 μg.ml^−1^ RNaseA (Sigma-Aldrich), and incubated for 45 min at 37°C, protected from light. The cells were then passed through a 35 μm nylon mesh cell strainer (BD Biosciences) and analysed using a BD FACSCalibur™ system (BD Biosciences). Data was acquired from the FSC, SSC, FL2-W and FL2-A detectors using BD CellQuest™ software (BD Biosciences), and further analysed and graphically represented using the ©FlowJo software, version 10. To assess mRNA knockdown efficiency, the levels of mRNA were analysed by quantitative real-time PCR (RT-qPCR). Briefly, ∼2 × 10^7^ cells were collected and total RNA extracted using the RNeasy Mini Kit (Qiagen), according to the manufacturer instructions. Next, 1 μg of total mRNA was converted into cDNA using the SuperScript™ III Reverse Transcriptase Kit (Life Technologies), according to the manufacturer. For the RT-qPCR reaction, primers were designed using the Primer Express version 3.0 software (Bio Rad), and according to established guidelines (61,62): TbORC4—AGGCAGTGAAGTCATTGTGG and AAGCGCGTAATTCCTGAGAG; TbORC1B—ACGTCAACTGTGCGGATATG and TCCAAGCGAACCTGTGAAC; Tb3120—GCTGCTTTGCAGGAAATACC and GCAGTGAAATGCTTCTGCTG. Primer sequences targeting TbORC1/CDC6 and the Tb927.10.12970 gene (here used as the endogenous reference gene for normalization) have been published previously (49,63). For each pair of primers, triplicates of each sample cDNA (diluted 1:10) were run per plate (MicroAmp® Optical 96-well Reaction Plate, Applied Biosystems®, Life Technologies), using Precision™ qPCR MasterMix with SYBR Green and low ROX (Primerdesign), as recommended by the manufacturer. The qPCR reactions were performed on a 7500 Real Time PCR system (Applied Biosystems®), using the following PCR cycling conditions: 95°C for 10 min, followed by 40 cycles of 95°C for 15 s and 60°C for 1 min. Fluorescence intensity data was collected at the end of the extension step (60°C for 1 min), and analysed by relative quantification using the ΔΔCt method (64), and the non-induced sample (Tet-) as the calibrator, on the 7500 software version 2.3 (Applied Biosystems®).

### RNA interference analysis in BSF cells

RNAi analysis in BSF cells was performed using a strategy described previously (65). Here, each gene fragment was PCR-amplified using a pair of primers containing the *att*B1 (forward primer) and *att*B2 sites, and cloned into the pGL2084 plasmid vector, in a site-specific BP recombination reaction, using the Gateway® BP clonase® II Enzyme mix Kit (Life Technologies), as described by the manufacturer. The following primers were used: TbORC1/CDC6—GGGGACAAGTTTGTACAAAAAAGCAGGCTGAAGCCCACAGCTGTCTTTC and GGGGACCACTTTGTACAAGAAAGCTGGGTTTCTCCGGCAACTTGTAACC; GGGGACCACTTTGTACAAGAAAGCTGGGTTTCAGTTTCGCCGAAGTTCT; TbORC1B—GGGGACCACTTTGTACAAGAAAGCTGGGTTTTGCGATTTGACTGTCTCG and GGGGACAAGTTTGTACAAAAAAGCAGGCTCTATCGGCTGAGTACGCCTC. The resulting constructs were then digested with AscI, transfected into BSF Lister 427 cells, strain 2T1, with TbORC1/CDC6 or TbORC1B endogenously tagged with 12myc at the C-terminus (as described in a previous section). Transformant cells were selected with 5 μg.ml^−1^ hygromycin, in the presence of 2.5 μg.ml^−1^ phleomycin and 10 μg.ml^−1^ blasticidin, and tested for susceptibility to puromycin (0.2 μg.ml^−1^). For RNAi analysis, each cell line was diluted into two different cultures of 1 × 10^4^ cells. ml^−1^, in HMI-11 thymidine-free media. To one of these, 1 μg.ml^−1^ tetracyclin (Calbiochem®, Merck Millipore) was added (Tet + sample) to induce the gene-specific RNAi, while the other culture was used as the non-induced (Tet-) sample. The cell density in both cultures was then assessed using a Neubauer improved hemocytometer. At each time point, 3 ml of each culture was collected for EdU incorporation and cell cycle (DAPI staining of nuclear and kinetoplastid DNA) analysis (described in specific sections). RNAi efficiency was assessed by western blot (below).

### EdU incorporation and quantification in PCF and BSF cells

Exponentially growing PCF cells were incubated with 50 μM of 5-ethynyl-2′-deoxyuridine (EdU; Life Technologies) for 3 h at 27°C, while BSF cells were incubated with 150 μM of EdU for 4 h at 37°C and 5% CO_2_. The cells were then collected and centrifuged at 1620 g (PCF) or 1000 g (BSF) for 10 min, washed once in 1x PBS (pH 7.2), and the pellet re-suspended in 200 μl of 1x PBS. Next, 20 μl of the cell suspension was loaded onto each well of a 12 multi-well glass slide (Thermo Scientific) previously treated with Poly-L-lysine (Sigma-Aldrich), and allowed to settle for 4 min. The cells were then fixed for 15 min (PCF) or 5 min (BSF) with 3.7% paraformaldehyde (Sigma-Aldrich) in 1x PBS. The cells were washed three times, 5 min each, with 3% BSA (Sigma-Aldrich) in 1x PBS, and permeabilized for 20 min (PCF) or 10 min (BSF) with 0.2% Triton X-100 (Promega), diluted in 1x PBS. The cells were then washed twice with 3% BSA, and incubated with 25 μl of the Click-iT® EdU detection mix (Life Technologies) for 1 h, protected from light. The Click-iT® EdU detection mix was composed of 21.5 μl of 1x Reaction Buffer, 1 μl of copper sulphate (CuSO4), 0.06 μl (PCF cells) or 0.25 μl (BSF cells) of Alexa Fluor® 555, and 2.5 μl of 1x Reaction Additive. The cells were then washed 4–6 times with 3% BSA, after which Fluoromount G containing DAPI mounting media (Cambridge Bioscience, Southern Biotech) was added, the slide covered with a coverslip, and sealed with regular nail varnish. In the case of imaging both incorporated EdU and 12myc-tagged proteins, the cells were incubated for 1 h, protected from light, with 20 μl of mouse anti-myc clone 4A6 Alexa Fluor® 488 conjugate antiserum (Millipore), diluted 1:500 in 1% BSA in 1x PBS, and washed three times with 3% BSA, before adding the mounting media. Images were acquired using a Zeiss Axioskop 2 fluorescent microscope (63x oil 1.4 NA objective) attached to an HBO100 lamp and a digital ORCA-ER camera and camera controller (Hamamatsu Photonics), using the Volocity® 6.1.1 Cellular and Imaging Analysis software (Perkin Elmer). Images were further analysed using Fiji (66). The numbers of EdU-positive and EdU-negative cells were assessed using the Cell Counter plugin, and the percentage of EdU-positive cells was calculated for each sample (Tet- and Tet+) per time point. The percentage of EdU-positive cells was then calculated relative to the percentage of the Tet- sample (considered to be 100%). Likewise, the number of cells in the different cell cycle stages was also assessed according to the nucleus-kinetoplast ratio, and the percentage of each cell cycle stage was then calculated to the total sampled population.

### Immunofluorescence of 12myc-tagged proteins in PCF and BSF cells

Approximately 1 × 10^7^ cells were collected from an exponentially growing PCF cell culture (∼1 × 10^7^ cells.ml^−1^), centrifuged for 10 min at 1620 g, and washed once in 1x PBS. The pellet was then re-suspended in 200 μl of 1x PBS, and 20 μl of cell suspension was loaded onto each well of a 12 multi-well glass slide (Thermo Scientific), previously coated with Poly-L-lysine (Sigma Aldrich), and allowed to settle for 4 min. Next, the cells were incubated for 15 min with 20 μl of 3.7% paraformaldehyde in 1x PBS, further washed three times, for 5 min each, with 20 μl of 3% BSA (Sigma Aldrich) in 1x PBS, and then incubated for 20 min with 20 μl of 0.2% Triton X-100 (Promega), diluted in 1x PBS. Subsequently, the wells were washed twice with 20 μl of 3% BSA in 1x PBS, after which each well was incubated for 1 h, protected from light, with 20 μl of mouse anti-myc clone 4A6 Alexa Fluor® 488 conjugate antiserum (Millipore), diluted 1:500 in 1% BSA in 1x PBS. Next, each well was washed three times with 3% BSA in 1x PBS, after which Fluoromount G containing DAPI mounting media (Cambridge Bioscience, Southern Biotech) was added, the slide covered with a coverslip, and sealed with regular nail varnish. In the case of BSF cells, ∼2 × 10^6^ cells were collected from an exponentially growing culture (∼1 × 10^6^ cells.ml^−1^), and centrifuged for 5 min at 1000 g. The cells were then washed in 1x PBS, and re-suspended in 50 μl of 1x PBS, to which 250 μl of 3.7% paraformaldehyde (Sigma-Aldrich) were then added, and incubated for 5 min. Next, 10 ml of 1x PBS was added, and the cells centrifuged for 5 min at 1000 g. The cells were then re-suspended in 20 μl of 1x PBS, and loaded onto each well of a 12 multi-well glass slide (Thermo Scientific), previously coated with Poly-L-lysine (Sigma Aldrich), and let to settle for 5 min. The cells were permeabilized with 20 μl of 0.2% Triton X-100 (Promega) for 10 min, after which the cells were treated as described above for PCF cells. For the quantification of the signal intensity of each 12myc-tagged protein throughout the cell cycle, images were acquired with a Zeiss Axioskop 2 fluorescent microscope (63x oil 1.4 NA objective) attached to an HBO100 lamp and a digital ORCA-ER camera and camera controller (Hamamatsu Photonics), using the Volocity® 6.1.1 Cellular and Imaging Analysis software (Perkin Elmer), and further analysed with Fiji (66). The number of cells containing myc signal was assessed using the Cell Counter plugin. In the case of measuring the intensity of the detected fluorescent signal (DAPI and myc), each image was treated using the Rolling Ball background subtraction plugin, set up with a radius of 50 pixels, and a circular 21 × 21 pixel region of interest (ROI) was drawn around each individual cell nucleus, and the mean pixel intensity within the ROI was measured. Images of both PCF and BSF cells were also obtained using a 100x oil immersion 1.4 NA objective on an DeltaVision Core microscope (Image Solutions, UK), equipped with a CoolSNAP HQ2 CCD camera (Photometrics®). Z-stacks of 5 μm thick (25 sections, 0.2 μm thickness each) were acquired with a 512 × 512 resolution using the SoftWoRx suite 2.0 software (Image Solutions, UK), and deconvolved using the ratio conservative method applied by the SoftWoRx software. These were further analysed with Fiji (66).

### Super-resolution imaging and analysis

Super-resolution Structured illumination (SR-SIM) microscopy was performed using an Elyra PS.1 super-resolution microscope equipped with a sCMOS PCO camera (Zeiss, Germany). The Plan-Apochromat 100x oil 1.46 NA objective was used, and Z-stacks of 4 μm thick (45 sections of 0.09 μm thickness each) were acquired using the ZEN Black Edition Imaging software (Zeiss, Germany). Five-phase SR-SIM images were reconstructed in the same software using the Structural Illumination manual processing tool. Maximum projection SR-SIM images were processed in their final form using FIJI (66) and Adobe Photoshop.

### Gel filtration

A HiLoad 16/60 Superdex 200 Prep Grade (GE Healthcare Life Sciences) column, set up in a ÄKTApurifier system (GE Healthcare Life Sciences), and controlled with Unicorn 5.31 software (GE Healthcare Life Sciences), was used. Prior to sample injection, the column was equilibrated with two column volumes of running buffer (50 mM of HEPES pH 7.55, 100 mM of NaCl, 1 mM of EDTA pH 8, 1 mM of EGTA pH 8, 10% Glycerol, 1% Triton X-100, 1 mM of DTT and 0.25x complete protease inhibitor cocktail), using a flow rate of 750 μl per min. Around 7.5 × 10^8^ cells were collected from an exponentially growing PCF cell culture (∼1 × 10^7^ cells.ml^−1^), centrifuged at 1620 g for 10 min and washed in 10 ml of 1x PBS. The pellet was then re-suspended in 2 ml of lysis solution (50 mM of HEPES pH 7.55, 100 mM of NaCl, 1 mM of EDTA pH 8, 1 mM of EGTA pH 8, 10% Glycerol, 1% Triton X-100, 1 mM of DTT and 2x complete protease inhibitor cocktail), and incubated on ice for 30 min. Two millilitre of lysate were then transferred into a 2.2 ml thin wall propylene centrifuge tube (11 × 35 mm) (Beckman Coulter), and centrifuged at 100 000 g for 1 h, at 4°C, using an Optima™ TL Ultracentrifuge (Beckman Coulter) equipped with a TLS 55 rotor (Beckman Coulter). The lysate was then filtered (0.2 μm Ministart® Syringe Filter, Sartorius), and injected into the ÄKTApurifier system, and run in a total of 210.8 ml of running buffer at a flow rate of 500 μl.min^−1^. Fractions of 1 ml were collected into ABgene 2.2 ml 96-well storage plates (Thermo Scientific) for further analysis by western blotting (below).

### Fluorescence-activated cell sorting (FACS)

Approximately 1 × 10^9^ cells were collected from an exponentially growing PCF culture (∼1 × 10^7^ cells.ml^−1^), centrifuged for 10 min at 1620 g and washed in 10 ml of 1x PBS supplemented with 5 mM of EDTA. The cells were then re-suspended in 12 ml of 1x PBS supplemented with 5 mM of EDTA, to which 28 ml of 100% ice cold-Methanol was added, in a drop-wise fashion while vortexing (final fixing solution was of 70% (v/v) methanol and a cell concentration of 2.5 × 10^7^ cells.ml^−1^). The cells were then kept at 4°C from overnight up to three weeks. For every FACS run, four FACS tubes (Becton Dickinson) were prepared, each starting with 4 ml of fixed cells (∼1 × 10^8^ cells). The cells were collected and centrifuged for 10 min at 1620 g, at 4°C, washed in 1 ml of 1x PBS supplemented with 5 mM of EDTA, re-suspended in 4 ml of 1x PBS supplemented with 5 mM of EDTA, 10 μg.ml^−1^ of PI (Sigma Aldrich) and 10 μg.ml^−1^ of RNase A (Sigma Aldrich), and incubated for 45 min at 37°C, protected from light. The cells were then transferred to a FACS tube through a cell strainer cap (BD Biosciences), and sorted into G1, early S, late S and G2 phases using a BD FACSAria I™ Cell Sorter (BD Biosciences). The sorted cells were collected at 4°C into new FACS tubes containing 200 μl of 1x PBS supplemented with 5 mM of EDTA. The collected cells were then centrifuged at 2000 g for 10 min, and re-suspended in 10 μl of 1x NuPAGE® LDS Sample Buffer (Life Technologies), and used for western blot analysis.

### Western blot

Approximately 2.5 × 10^6^ PCF or BSF cells were collected for immunodetection of the proteins of interest by western blot. The 12myc-tagged proteins were detected using the mouse anti-myc clone 4A6 antiserum (diluted 1:7000; Millipore), while 6HA-tagged proteins were detected using the mouse anti-HA clone HA-7 antiserum (diluted 1:10 000; Sigma-Aldrich). Whenever necessary, the transcription elongation factor Ef1α was used as a loading control and detected with the mouse anti-Ef1α clone CBP-KK1 antiserum (diluted 1:25 000; Millipore). The three antisera were used in combination with the goat anti-mouse IgG (H+L) horseradish peroxidase conjugate (diluted 1:5000; Life Technologies).

### Statistical analysis

All statistical analyses were performed using Prism 6 (GraphPad software Inc.), and the statistical tests used are described in the figure legend of the corresponding graph.

### Protein sequence analysis and structure prediction

Gene and protein sequences (TbORC1/CDC6 – Tb927.11.7216; TbORC1B – Tb927.9.2030; TbORC4 – Tb927.10.13380; Tb3120 – Tb927.9.4530; and Tb7980 – Tb927.10.7980) were retrieved from the TriTrypDB online database (http://tritrypdb.org/tritrypdb/). For protein homology searches, protein sequences were analysed using the standard (default settings) protein–protein Basic Local Alignment Search Tool (BLAST) (blastp) (http://blast.ncbi.nlm.nih.gov/Blast.cgi), with the non-redundant protein sequences database (nr). For protein structure and function prediction, the RaptorX (67) online server was used. Protein alignments were performed using CLC genomics Workbench, with a gap open cost of 10.0, gap extension cost of 1.0, end gap cost of ‘as any other’, and the very accurate (slow) option for alignment. Protein domain searches were performed using Pfam, version 27.0 (http://pfam.xfam.org/), using default settings. Figures representing the predicted structure of Tb3120 and Tb7980, as well as the structures of *D. melanogaster* Orc2 and Orc5 (imported from RCSB Protein Bank Database) subunits, were generated in CLC genomics Workbench.

## RESULTS

### TbORC4 and Tb3120, two TbORC1/CDC6-interacting factors, contribute to nuclear DNA replication

**Figure 1.**
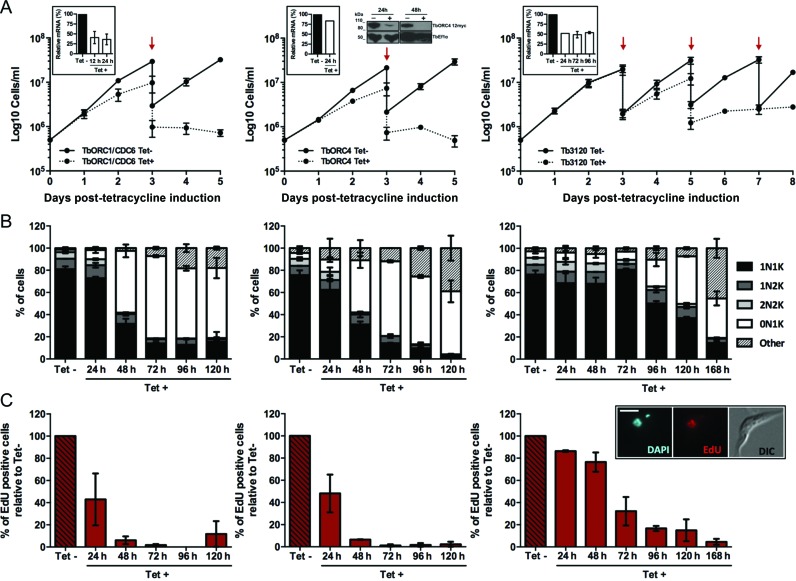
Effect of induced RNAi against TbORC1/CDC6, TbORC4 or Tb3120 in PCF *T. brucei*. (**A**) Growth curves of uninduced (Tet-) and tetracycline-RNAi induced (Tet+) cell cultures over 5 (TbORC1/CDC6 and TbORC4) or 7 days (Tb3120). Cell concentration was assessed every 24 h and the mean concentration from two independent experiments is shown; error bars depict the standard error of the mean (SEM). Red arrows denote times at which a 1:10 dilution of the Tet- or Tet+ cultures was carried out at selected time points after RNAi induction. Insert box: efficiency of RNAi knockdown of mRNA levels assessed by RT-qPCR at selected time points after RNAi induction. The results represent the levels of mRNA in the Tet+ sample relative to non-induced, calculated using the ΔΔC_t_ method. The mean of two independent experiments is shown, and error bars represent SEM. For TbORC4, the levels of the endogenously 12myc-tagged TbORC4 were assessed by western blot analysis of whole cell extracts 24 and 48 h after RNAi induction (+) or without induction (-); *T.brucei* Elongation factor 1 α (Ef1α) was used as a loading control. (**B**) Quantification of the proportion of different cell types throughout the course of the RNAi induction, based on the number of nuclei (N) and kinetoplasts (K) detected in individual cells stained with DAPI. A minimum of 150 cells were counted per time point and experimental group (Tet- and Tet+), and percentages of each cell type (1N1K, 1N2K, 2N2K, 0N1K and others) were calculated relative to the total amount of cells analysed. The graph represents the mean of each cell type observed in two independent experiments, while the error bars show SEM. (**C**) Percentage of cells displaying nuclear EdU signal in the Tet+ samples relative to the number of EdU positive cells in the Tet- culture from the same time point. A minimum of 150 cells were analysed per time point and group (Tet- and Tet+) and the mean from two independent experiments is shown; error bars represent the SEM. The insert in the Tb3120 column shows an example of an EdU positive cell, relative to the same cell DAPI-stained and DIC imaged; scale bar represents 5 μm.

To facilitate gene function analysis, both PCF and BSF life cycle stages of *T. brucei* have been genetically modified to allow tetracycline (Tet) inducible expression of gene-specific dsRNA, allowing controlled activation of RNAi against any gene. Two different strategies to express gene-specific dsRNA from the *rRNA* locus have been adopted: insertion of a target gene fragment between opposing Tet-controlled promoters (68,69) and insertion of inverted repeats of a gene fragment to generate stem-loop RNA from a single Tet-controlled promoter (70–72). Previously, we reported RNAi against TbORC1/CDC6, TbORC4, Tb7980 and Tb3120 in PCF cells by the former approach, which resulted in remarkably mild growth and cell cycle defects, visible only >4 days post RNAi induction (49). Perhaps due to the weakness of these RNAi effects, testing if loss of these factors caused impaired nuclear DNA replication was inconclusive (49). Because RNAi mediated by stem-loop RNAs has been suggested to be more efficient, we sought to generate PCF RNAi cell lines for each of putative *T. brucei* ORC-like factors using stem-loop constructs. Figure 1 shows the effect of induced stem-loop RNAi against TbORC1/CDC6, TbORC4 and Tb3120. Unfortunately, we were unable to generate a stem-loop RNAi cell line targeting Tb7980, for reasons that remain unclear.

Tet-induced RNAi against TbORC1/CDC6 or TbORC4 resulted in strikingly similar phenotypes. For both factors, a growth defect (Figure 1A) was seen from 48 h after RNAi induction, with growth slowing until ∼96 h and subsequent cell death. Concomitant with growth impairment, aberrant cells were detected by DAPI staining (Figure 1B). In *T. brucei* the nuclear (N) and kinetoplastid (K) genomes possess distinct S phase timing, with replication and segregation of the latter being complete before the former (73). Therefore, analysis of the ratio and morphology of the nucleus and kinetoplast delineates the cell cycle stage of individual cells in an asynchronous population: most 1N1K cells are in G1 phase; 1N1eK (elongated—replicating—but not yet divided kinetoplast) are in S phase; 1N2K cells are in S-G2 phase and 2N2K cells are post-mitotic (74). A dramatic increase in numbers of enucleated cells (0N1K or zoids) (75) was seen after RNAi for both genes: zoids represented only ∼1–3% of the populations prior to RNAi induction, but increased to 50–60% 48 h post-induction and reached a maximum of ∼70% at ∼72 h (Figure 1B). Concurrently, the numbers of 1N1K cells decreased from ∼80% of the uninduced populations to ∼15% from 72 h onwards, and 2N2K cells were virtually abolished from both populations by 72 h post-induction. Though increased levels of 1N2K cells might be expected if replication was affected and a G2/M checkpoint was enacted, there was little evidence for this; indeed, if anything, levels of 1N2K cells decreased. At late time points some further abnormal cells could be seen beyond the predominant zoids; we have referred to these cells as ‘others’ because no predominant N-K ratio could be discerned and they frequently displayed an irregular cellular shape (Supplementary Figure S1). The cell cycle effects following RNAi were supported by flow cytometry analysis of PI-stained cells (Supplementary Figure S2): from 48 h onwards, an increase in a sub-G1 population (<2n DNA content, suggesting lack of a nucleus) was observed, in parallel with a decrease in G1 (2n) and G2/M (4n) cells, which by 96 h after RNAi induction was profound. The levels of 0N1K cell accumulation presented here exceed that described in previous RNAi studies on these factors (49,53), as does the timing of onset and severity of the growth defects, perhaps suggesting that the RNAi approach was more effective, despite the fact that quantitative RT-qPCR suggested only a ∼60% reduction in TbORC1/CDC6 mRNA 24 h after RNAi induction (Figure 1A). Indeed, since RT-qPCR suggested a very modest loss of TbORC4 mRNA at the same time point (∼20%), TbORC4 was endogenously tagged at the C-terminus with 12 copies of the myc epitope (see below) in the RNAi cell line targeting TbORC4, revealing that the protein was undetectable 48 h after RNAi induction (Figure 1A).

RNAi against Tb3120 resulted in a later and milder growth defect than seen for TbORC1/CDC6 or TbORC4, only apparent ∼96 h after induction (Figure 1A). Nonetheless, like for TbORC1/CDC6 and TbORC4, the growth defects following Tb3120 RNAi correlated with the accumulation of aberrant cells (initially zoids and, later, ‘others’) and increasing loss of 1N1K and 2N2K cells in the population (Figure 1B), suggesting a comparable RNAi response. Flow cytometry analysis confirmed these observations, as sub-G1 cell accumulation was detected from 96 h after RNAi induction, and G1 and G2 cells were strongly reduced by 168 h (Supplementary Figure S2). Why the RNAi phenotypes accumulated more slowly upon Tb3120 depletion is unclear, since RT-qPCR suggested similar levels of Tb3120 mRNA loss (∼50% at 24, 48 and 96 h) to those seen for TbORC1/CDC6 (Figure 1A).

Recently, loss of TbORC1/CDC6 has been shown to impair DNA replication, since a decrease in the levels of bromo-5′-deoxyuridine (BrdU) incorporation was detected after RNAi induction (54). To corroborate these observations, and to extend them to the TbORC1/CDC6-interacting factors, we evaluated uptake of 5-ethynyl-2′-deoxyuridine (EdU; which is also an analogue of thymidine) (76) after TbORC1/CDC6, TbORC4 or Tb3120 RNAi (Figure 1C). For this assay, both non-induced and induced cells were incubated with EdU for 3 h and uptake examined by fluorescent microscopy (Figure 1C, insert box). The percentage of EdU-labelled cells in the RNAi-induced sample was calculated relative to the number of EdU-positive cells in the non-induced sample (Figure 1C). For all three factors, the number of EdU-positive cells decreased with time in the RNAi induced cells relative to the uninduced, and the extent of the loss mirrored the severity of the growth and cell cycle phenotypes, with earlier loss after TbORC1/CDC6 or TbORC4 RNAi (both reduced by ∼50% at 24 h) than for Tb3120 (∼60% reduction at 72 h) (Figure 1C). Importantly, in all cases the timing of EdU incorporation decrease preceded the appearance of growth or cell cycle defects, arguing that the aberrant cells are a consequence, not the cause, of impaired nuclear DNA replication.

### TbORC1B acts in *T. brucei* nuclear DNA replication

**Figure 2.**
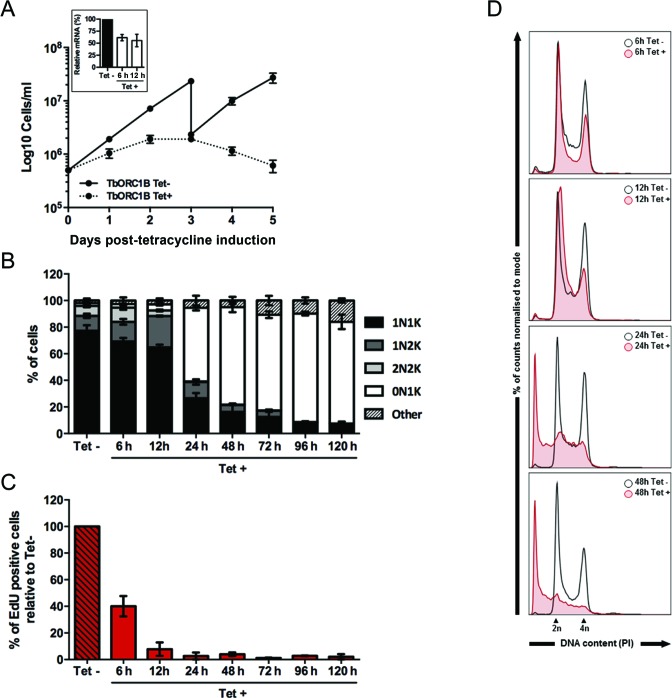
Effect of induced RNAi against TbORC1B in PCF *T. brucei*. (**A**) Growth curves of uninduced (Tet-) and tetracycline-RNAi induced (Tet+) cell cultures over five days, where cell concentration was assessed every 24 h; mean concentration from two independent experiments is shown and error bars depict the standard error of the mean (SEM). Insert box: efficiency of RNAi knockdown of TbORC1B mRNA levels assessed by RT-qPCR. The results represent the amount of mRNA at the time points shown after RNAi induction (Tet+) relative to the non-induced sample (Tet-). The mean of two independent experiments is shown, and the error bars represent SEM. (**B**) Quantification of the proportion of different cell cycle types throughout the course of the RNAi induction, based on the number of nuclei (N) and kinetoplasts (K) detected in individual cells stained with DAPI. A minimum of 150 cells were counted per time point and experimental group (Tet- and Tet+), and percentages of each cell type (1N1K, 1N2K, 2N2K, 0N1K and others) were calculated relative to the total amount of cells analysed. The graph represents the mean of each cell type observed in two independent experiments, while the error bars show SEM. (**C**) Percentage of cells displaying nuclear EdU signal in the Tet+ samples relative to the number of EdU positive cells in the Tet- culture from the same time point. A minimum of 150 cells were analysed per time point and group (Tet- and Tet+) and the mean from two independent experiments is shown; error bars represent the SEM. (**D**) Histograms representing the distribution of the cell population according to DNA content (stained with PI) assessed by flow cytometry at the 6, 12, 24 and 48 h time points. Approximately 30 000 cells were analysed per sample, and the histograms represent the percentage of cells in the population, normalized to mode; cells with 2n and 4n DNA content are indicated.

TbORC1B RNAi silencing, which has not to date been reported, resulted in the most severe growth defect of all the factors tested, evident as early as 24 h post-induction (one day before any growth effect was observed following TbORC1/CDC6 or TbORC4 RNAi; Figure 2A). Due to this rapid response, cell cycle and EdU incorporation analyses were conducted at earlier time points. Six hours after RNAi induction, when RT-qPCR showed ∼40–50% loss of TbORC1B mRNA (Figure 2A), there was no increase in the proportion of zoids or other aberrant cells (below), but a small increase in 1N2K and 2N2K cells was observed, together with a decrease in 1N1K cells (Figure 2B). Twelve hours after RNAi induction, a small increase in zoids was noticeable, together with a pronounced increase in 1N2K cells and decrease in both 1N1K and 2N2K cells, suggesting an accumulation of cells in G2/M phase and a decrease in G1 phase cells. However, this was not supported by flow cytometry analysis, where an increase in the G1 (2n) population and a decrease in the G2/M (4n) population was observed instead (Figure 2D). Quantification of EdU labelling revealed ∼60 and ∼90% reduction in EdU positive cells by 6 and 12 h post-induction (Figure 2C), respectively, indicating that cells morphologically classified as 1N2K at these time points have not replicated their nuclear DNA, although the kinetoplast has replicated and segregated as normal. As for the other factors, reduced EdU incorporation preceded the emergence of aberrant cells: at 24 h post-induction, when EdU incorporation was essentially undetectable (Figure 2C), >55% of the population was composed of zoid cells (Figure 2B), while both 1N1K and 1N2K populations were reduced, and virtually no 2N2K cells were observed, mirroring the cell cycle phenotypes seen at ∼48 h post RNAi for TbORC1/CDC6 or TbORC4. These cell cycle defects were reflected in the flow cytometry analysis at 24–48 h post RNAi, which showed a pronounced reduction in both G1 (2n) and G2/M (4n) cell populations, and an increase in the levels of sub-G1 cells (Figure 2D). Between 48 and 72 h post-induction cell growth stalled (Figure 2A) and thereafter cell numbers decreased, which was accompanied by an increase in the number of zoids to ∼70–80% of the population (Figure 2B), in parallel with a further decrease in 1N1K and 1N2K cells, complete loss of 2N2K cells and a small increase in ‘others’ (reaching ∼15% at the latest time point, 120 h).

### TbORC1B differs from all other identified TbORC1/CDC6 interacting factors in displaying cell cycle regulated expression

Both *T. brucei* and *T. cruzi* ORC1/CDC6 proteins have been reported to localize to the nucleus and associate with chromatin throughout the cell cycle of replicating cells (53,77). To compare this localization pattern with the TbORC1/CDC6 interacting factors, each protein was tagged at its endogenous locus with 12 copies of the c-myc epitope (12myc), either at the C-terminus (TbORC4, TbORC1B and Tb3120), as described previously for TbORC1/CDC6 (49), or at the N-terminus (Tb7980), using a modified version (gift, A. Trenaman) of the pEnT6B construct (59). Expression of the tagged proteins in PCF cells was confirmed by western blot (Supplementary Figure S3A), and no cell growth (Supplementary Figure S3B), cell cycle (Supplementary Figure S3C) or EdU uptake (Supplementary Figure S3D) defects were detected in these cells compared with the parental cell line (927 wt) or the TbORC1/CDC6–12myc expressing cells (55).

**Figure 3.**
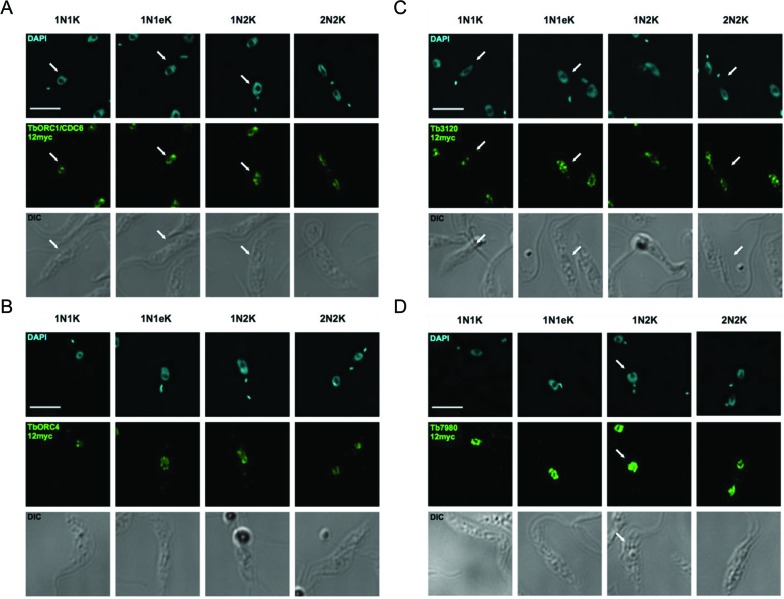
Immunofluorescence localization of TbORC1/CDC6, TbORC4, Tb3120 and Tb7980 in PCF *T. brucei* cells. Analysis of the subcellular localization of 12myc tagged variants of TbORC1/CDC6 (**A**), TbORC4 (**B**), Tb3120 (**C**) and Tb7980 (**D**) are shown in fixed cells. In each case the cells are shown stained with DAPI, allowing their classification as 1N1K cells (G1 phase), 1N1eK cells (S phase), 1N2K cells (G2/M phase) and 2N2K cells (post-mitosis). The tagged proteins are detected in each cell type using an anti-myc antiserum coupled with the Alexa Fluor 488 flurophore (myc, green). Finally, the outline of all cells is shown by DIC imaging. Arrows direct the reader's attention to single cells of the expected cell cycle stage if more than one cell is shown. Images were acquired using a DeltaVision imaging system and deconvolved using the ratio conservative method, on SoftWoRx software. The scale bar represents 5 μm.

As expected (53), TbORC1/CDC6–12myc localized to the nucleus of the cell throughout the cell cycle, displaying a punctate signal throughout most of the nucleoplasm, but apparently excluded from the nucleolus (Figure 3A). Unlike ORC1/CDC6 in *T. cruzi* epimastigotes (78), TbORC1/CDC6–12myc did not re-localize to the nuclear periphery during S phase; instead, TbORC1/CDC6–12myc distribution was indistinguishable in 1N1K, 1N1eK, 1N2K or 2N2K cells. Localization of TbORC4–12myc (Figure 3B), Tb3120–12myc (Figure 3C) and 12myc-Tb7980 (Figure 3D) showed that each of these factors behaves like TbORC1/CDC6–12myc: all localized, as puncta, to the nucleus of the cell throughout the cell cycle.

**Figure 4.**
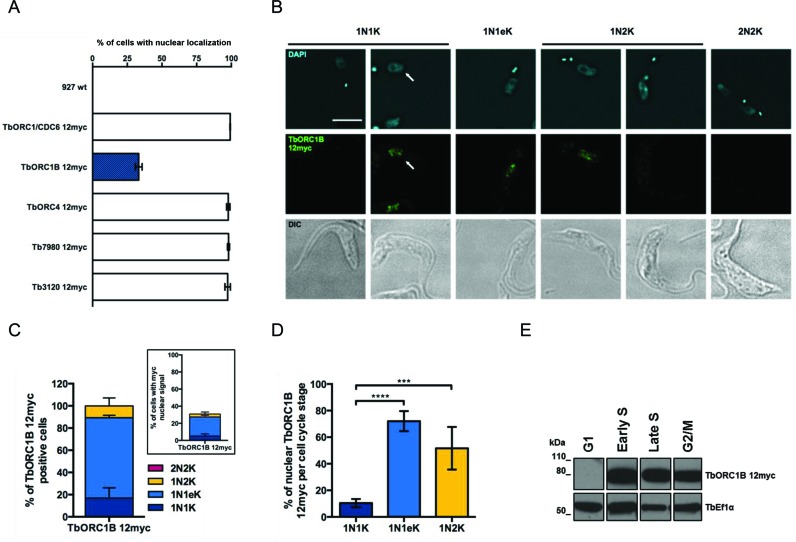
Cell-cycle dependent nuclear localization of TbORC1B. (**A**) Percentage of cells containing nuclear myc signal in untagged *T. brucei* PCF cells (927 wt) relative to PCF cells expressing TbORC1/CDC6–12myc, TbORC1B-12myc, TbORC4–12myc, 12myc-Tb7980 or Tb3120–12myc, each from the endogenous locus; the mean of three independent experiments is shown (>125 cells each) and error bars show SEM. (**B**) Immunofluorescent detection of TbORC1B-12myc with anti-myc antiserum (middle row) in 1N1K cells (G1 phase), 1N1eK cells (S phase), 1N2K cells (G2/M phase), and 2N2K cells (post-mitosis), stained with DAPI (top row) and cell outline shown by DIC (lower row). Arrows highlight a single cell of the expected cell cycle stage if more than one cell is captured in the images. The scale bar represents 5 μm. (**C**) The proportion of cell cycle stages displaying TbORC1B-12myc signal is shown either as the percentage of total cells (insert box), or as the percentage of positive cells (main graph), as determined by N-K ratio (dark blue, 1N1K cells; light blue, 1N1eK cells; yellow, 1N2K cells, red, 2N2K). The mean is shown from two independent experiments, and the error bars depict standard deviation. (**D**) Percentage of individual cell cycle stage cells that display TbORC1B-12myc signal (as no 2N2K cells were detected to have TbORC1B-12myc signal, these are not represented); the mean of four independent experiments is represented and error bars show standard deviation. Statistical significance between the different groups was assessed using the one-way ANOVA parametric test: (***) *P*-value < 0.001; (****) *P*-value <0.0001. (**E**) Asynchronous TbORC1B-12myc expressing cells were separated into G1, Early S, Late S and G2/M phases FACS sorting and the western blot shows the sorted fractions probed with anti-myc antiserum (α-myc); the same blot was probed with antiserum against the transcription elongation factor Ef1α as a loading control.

TbORC1B-12myc localization was strikingly different from all the above factors. TbORC1B-12myc was detected in the nucleus of only ∼33% of cells (Figure 4A and B), in contrast to TbORC1/CDC6–12myc, TbORC4–12myc, Tb3120–12myc and 12myc-Tb7980, where signal was detected in 100% of the cells (Figure 4A). Categorization of the N-K ratio of the cells showed that TbORC1B-12myc signal was not evenly distributed amongst the identifiable cell cycle phases (Figure 4B and C): most TbORC1B-12myc signal (∼72.5%) was found in 1N1eK cells, while the remainder was observed in both 1N1K and 1N2K cells (∼16.8 and ∼10.7%, respectively). Quantifying each cell cycle phase (Figure 4D) showed that ∼10.4% of 1N1K cells, ∼72% of 1N1eK cells and ∼51.6% of 1N2K cells had detectable TbORC1B-12myc signal, while no signal was observed in cells undergoing mitosis (1N2K cells with elongated nucleus) or in post-mitotic 2N2K cells (Figure 4B). Taken together, these data suggest that TbORC1B-12myc is only detectable in the nucleus of cells spanning the period from late G1 (or early S) phase to either late S or G2 phase (Figure 4B). To test this further, TbORC1B-12myc cells in early-mid G1, early S, late S and G2/M phases were isolated by fluorescence-activated cell sorting (FACS) and analysed by western blotting. This revealed that TbORC1B-12myc was undetectable in G1 phase cells, but was present in the other sorted cell cycle stages (Figure 4E). Taken together, these data suggest that TbORC1B protein levels are limited in non-replicating cells.

**Figure 5.**
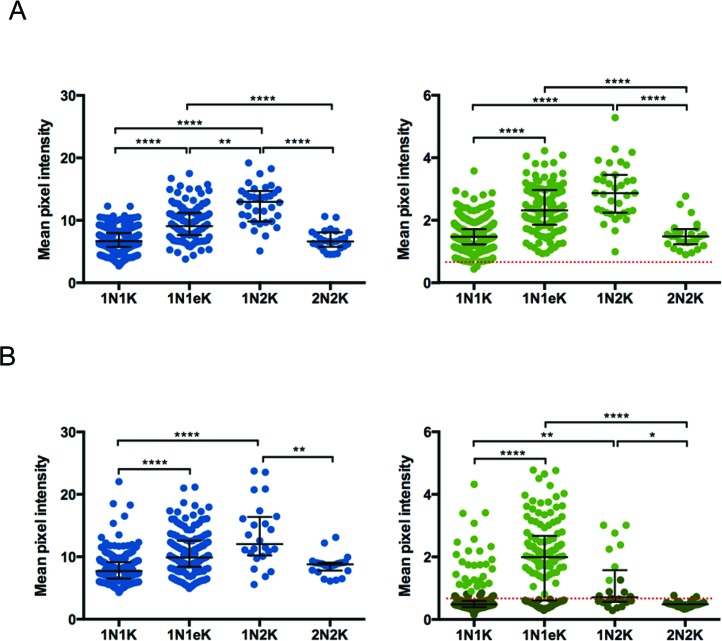
Quantification of TbORC1/CDC6 and TbORC1B subcellular localization through the cell cycle. Intensity of the DAPI (left graphs, blue dots) and myc signals (right graphs, green dots) of PCF *T. brucei* cells expressing TbORC1/CDC6–12myc (**A**) or TbORC1B-12myc (**B**) is represented as the mean pixel intensity within a circular ROI (21 × 21 pixels), drawn around each individual cell nucleus; for the myc signal data, the red dotted line represents the average background signal measured in 927 wt cells that do not express any tagged protein. Dark green dots represent cells in which TbORC1B-12myc signal was not visually detected, while light green dots represent cells with visually detectable TbORC1B-12myc signal. In each case, cells are separated by cell cycle stage, determined by N-K ratio in the DAPI images: 1N1K cells (G1 phase), 1N1eK cells (S phase), 1N2K cells (G2/M phase) and 2N2K cells (post-mitosis). The median values derived from the analysis of 591 TbORC1/CDC-12myc cells (1N1K – 1.47; 1N1eK – 2.31; 1N2K – 2.86; 2N2K – 1.48) and 412 TbORC1B-12myc cells (1N1K – 0.48; 1N1eK – 1.99; 1N2K – 0.71; 2N2K – 0.48) are represented, with error bars depicting the interquartile range. Statistical significance between the different cell cycle stages was assessed using the Kruskal-Wallis non-parametric test: (*) *P*-value < 0.05; (**) *P*-value < 0.01; (****) *P*-value < 0.0001.

Next, we measured the intensity of the DAPI and anti-myc fluorescence signals in the nuclei of individual myc-tagged cells, classified into the different cell cycle stages according to their N–K ratio. For all cell lines (Figure 5; Supplementary Figure S4), the DAPI fluorescence intensity increased as cells progressed from G1 phase (1N1K) through S (1N1eK), where cells showed a wider range of intensity values, most likely representing cells in the different points of S phase. The DAPI signal peaked in G2/M cells (1N2K), with approximately double the G1 signal, consistent with the DNA being completely replicated (Figure 5; Supplementary Figure S4). After mitosis (2N2K cells), the signal of each individual nucleus in the cell returned to intensity values observed in G1 cells. The intensity of TbORC1/CDC6–12myc closely followed the cell cycle dynamics of DAPI (Figure 5A), as did TbORC4–12myc, Tb3120–12myc and 12myc-Tb7980 (Supplementary Figure S4). These data suggest the abundance of these factors in the nucleus increases as the amount of DNA in the nucleus of the cell increases, peaking when the genome is completely replicated. Again, the data for TbORC1B-12myc differed (Figure 5B): for this factor anti-myc signal intensity peaked in 1N1eK cells, reduced in 1N2K cells and was at background levels in 2N2K cells. Indeed, the average signal in 1N1K cells was also at background levels, though some cells showed higher values, consistent with TbORC1B-12myc expression in only a fraction of this cell type. When taken together with the western blot data after FACS, it seems likely that TbORC1B-12myc expression is largely or completely limited to S-phase cells.

### Super-resolution imaging suggests related localization of TbORC1/CDC6, TbORC4 and TbORC1B

**Figure 6.**
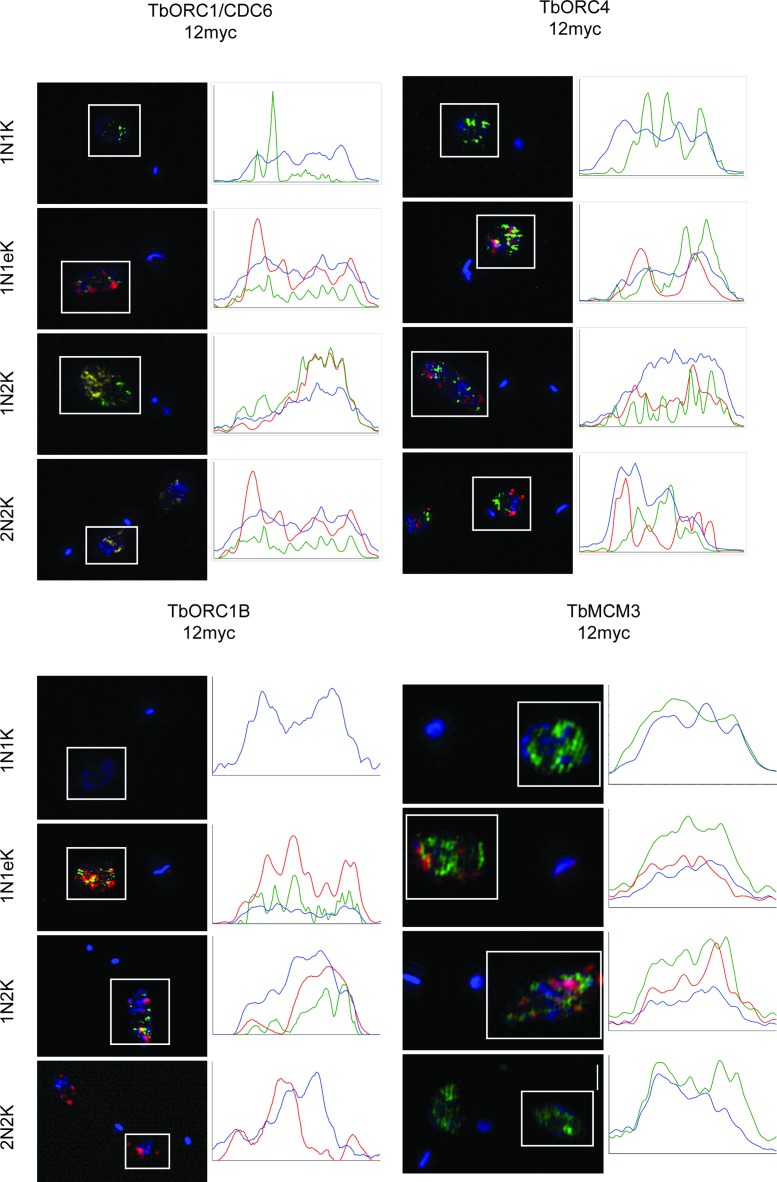
Super-resolution imaging of TbORC1/CDC6, TbORC4, TbORC1B and TbMCM3 through the cell cycle. PCF *T. brucei* cells expressing TbORC1/CDC6–12myc, TbORC4–12myc, TbORC1B-12myc or TbMCM3–12myc were incubated for 3 h with 150 μm EdU, fixed and stained with DAPI and with AlexaFluor® 488-conjugated anti-myc antibody, while EdU was detected with AlexaFluor® 594-conjugated azide. Images were acquired with a Zeiss Elyra super-resolution microscope system in SIM mode. In each case, representative maximum projection images are shown of cells in the different cell cycle stages, determined by N-K ratio in the DAPI images: 1N1K cells (G1 phase), 1N1eK cells (S phase), 1N2K cells (G2/M phase) and 2N2K cells (post-mitosis). All cells are shown as a merge of the EdU (red), anti-myc antiserum (green) and DAPI (blue) signals, whose intensities are quantified in the plots to the left; signal intensities (y-axes, arbitrary units) were analysed in a horizontal line across the boxed area surrounding the nucleus.

To compare in more detail the pattern of TbORC1/CDC-12myc, TbORC4–12myc and TbORC1B-12myc localization, we examined each using anti-myc immunofluorescence and super-resolution structure illumination microscopy (SR-SIM; Figure 6, and see Supplementary Figures S5–S8 for more detail). In addition, we compared the proteins’ localization with replicating DNA, which was detected with EdU. The use of SR-SIM allowed us to quantify the signal intensity and pattern within the nucleus, confirming that TbORC1/CDC6–12myc and TbORC4–12myc (Figure 6) do not localize homogenously in the nucleus, as the signals were present in discrete puncta in non-replicating cells (1N1K, 2N2K). There was no evidence for localization to any specific region within the nucleus. In early S phase cells (1N1eK) there was limited co-localization between either TbORC1/CDC6–12myc or TbORC4–12myc and EdU, which was also seen in puncta. However, overlap in the anti-myc and EdU signals became more pronounced in 1N2K cells, where S phase is more complete, and at that cell cycle stage the protein and replication puncta become more abundant and appeared more diffuse. As noted previously (79), there was no evidence that EdU signal accumulated in the periphery of the nucleus, reinforcing the suggestion of a difference in replication dynamics between *T. brucei* and *T. cruzi*. In 2N2K cells, after completion of replication, localization of TbORC1/CDC6–12myc and TbORC4–12myc returned to a comparable pattern to that of 1N1K cells. The SR-SIM imaging confirmed the cell cycle dependence of TbORC1B-12myc nuclear localization (Figure 6) and revealed a comparable pattern to TbORC1/CDC6–12myc and TbORC4–12myc in replicating cells (1N1eK and 1N2K), with signal again seen throughout the nucleus in a similarly large number of puncta and with some overlap with EdU. To ask to what extent the punctate signals are specific for the three ORC-related factors, we next performed SR-SIM imaging of PCF cells expressing a C-terminally tagged variant of the MCM helicase subunit 3 (TbMCM3–12myc) (49). Though there is evidence that TbMCM3 interacts with putative TbORC components (see below) (56), at least some of the MCM2–7 helicase is expected to move with the replisome and not therefore be limited to specific genomic sites, unlike TbORC1/CDC6 (55). Consistent with this suggestion, TbMCM3–12myc signal (Figure 6) was more abundant and more homogenous in the nucleus than any of the three ORC-related factors, and displayed little obvious variation in the different cell cycle stages, indicating that each of TbORC1/CDC6, TbORC4 and TbORC1B displays distinct sub-nuclear localization to that of TbMCM3.

### TbORC1B expression and function are conserved in mammal-infective *T. brucei* cells

**Figure 7.**
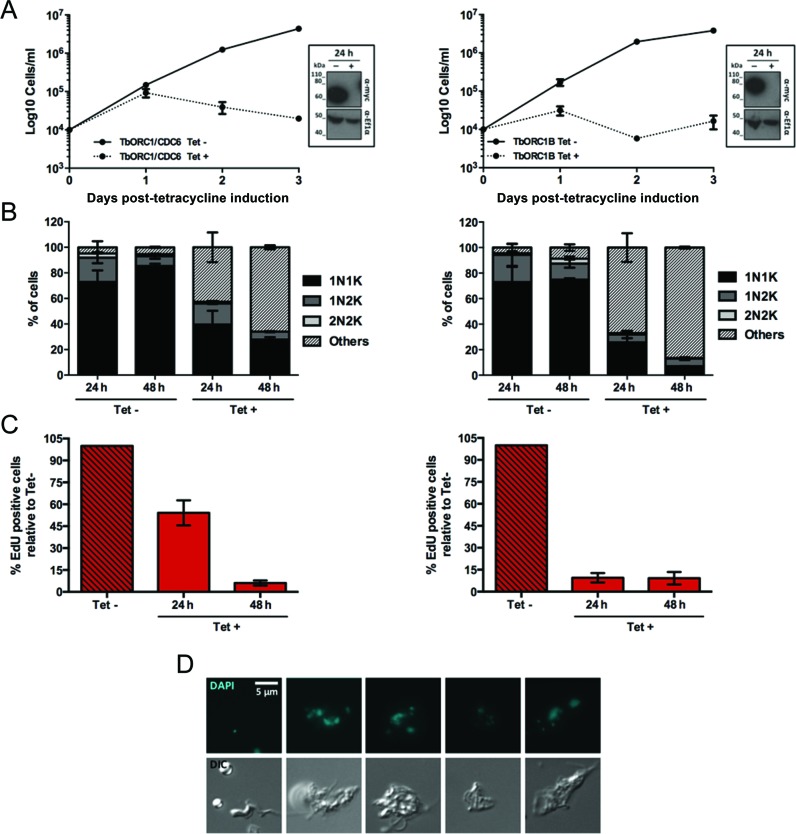
Effect of induced RNAi against TbORC1/CDC6 or TbORC1B in bloodstream form *T. brucei*. (**A**) Growth curves of uninduced (Tet-) and tetracycline-RNAi induced (Tet+) cells targeting TbORC1/CDC6 (left) or TbORC1B (right) are shown over 3 days. Cell concentration was assessed every 24 h and the mean concentration from two independent experiments is shown; error bars depict the SEM. Levels of 12myc-tagged protein are shown at 24 h time with (+) or without (-) RNAi induction by western blotting of whole cell extracts with anti-myc antiserum (α-myc); loading controls are shown by probing the same blot with antiserum against *T.brucei* Ef1α; size markers are indicated. (**B**) Quantification of the proportion of different cell types throughout the course of the RNAi induction, based on the number of nuclei (N) and kinetoplasts (K) detected in individual cells stained with DAPI. A minimum of 125 cells were counted per time point and experimental group (Tet- and Tet+), and percentages of each cell type (1N1K, 1N2K, 2N2K, 0N1K and others) were calculated relative to the total amount of cells analysed. The graph represents the mean of each cell type observed in two independent experiments, while the error bars show SEM. (**C**) Percentage of cells displaying nuclear EdU signal in the Tet+ samples relative to the number of EdU positive cells in the Tet- culture from the same time point. A minimum of 125 cells were analysed per time point and group (Tet- and Tet+) and the mean from two independent experiments is shown; error bars represent the SEM. (**D**) Examples of aberrant cells after TbORC1/CDC6 RNAi, stained with DAPI, and cell outline shown as a DIC image.

To ask if the function and cell cycle-dependent expression of TbORC1B in PCF cells is also seen in mammal-infective BSF cells, we generated stem-loop RNAi constructs (65) for the individual targeting of TbORC1/CDC6 or TbORC1B. The constructs were introduced into BSF 2T1-derived (80) cells expressing either TbORC1/CDC6 or TbORC1B endogenously tagged with 12myc. Twenty-four hours after RNAi induction, when TbORC1/CDC6–12myc or TbORC1B-12myc was no longer detectable by western blotting (Figure 7A, insert box), individual loss of the proteins resulted in cell death (Figure 7A). The greater rapidity and severity of growth impairment in BSF cells after TbORC1/CDC6 RNAi when compared with PCF cells is consistent with previous observations (49), despite the different RNAi strategy used here. Growth impairment was accompanied by a reduction in 1N1K, 1N2K and 2N2K cells and the accumulation of aberrant cells (Figure 7B), which were common to TbORC1/CDC6 and TbORC1B RNAi, but differed from those seen after PCF RNAi: 0N1K cells were detected, but were rare, and instead enlarged BSF cells with multiple nuclei and/or kinetoplasts predominated (examples in Figure 7D), consistent with previous reports of TbORC1/CDC6 RNAi in BSF cells (49). In common with the RNAi assays in PCF cells, aberrant cells arose more quickly and accumulated to a greater extent after TbORC1B RNAi-induction compared with TbORC1/CDC6, and this effect correlated with an earlier onset of loss of EdU incorporation (Figure 7C). Taken together, these data indicate that, like in PCF cells, the loss of either TbORC1/CDC6 or TbORC1B impairs nuclear DNA replication in BSF *T. brucei*.

To ask if the localization of TbORC1/CDC6 and TbORC1B, as well as the other TbORC1/CDC6-interacting factors, is conserved between PCF and BSF life cycle stages, all genes were 12myc-tagged in Lister 427 BSF cells and expression of the epitope tagged proteins confirmed (Supplementary Figure S10A). Immunofluorescence microscopy showed that TbORC1/CDC6–12myc (Supplementary Figure S9A) was, like TbORC4–12myc, Tb3120–12myc and 12myc-Tb7980 (Supplementary Figure S9B, C and E), detectable in the nucleus of all BSF cell cycle stages. In contrast, TbORC1B-12myc showed essentially the same cell cycle-dependent localization in BSF cells (Supplementary Figures S9D and S10B–D) as described in PCF cells, with nuclear signal being most pronounced in 1N1eK and 1N2k cells (∼90 and ∼50%, respectively), more limited in 1N1K cells (∼20%) and virtually undetectable in 2N2K cells (only one cell in >200 examined) (Supplementary Figure S10D). It seems likely, therefore, that TbORC1B expression is limited to S phase in all replicating *T. brucei* cell types.

### TbORC1/CDC6, TbORC4 and TbMCM3 are present in a common high molecular weight complex in *T. brucei*

**Figure 8.**
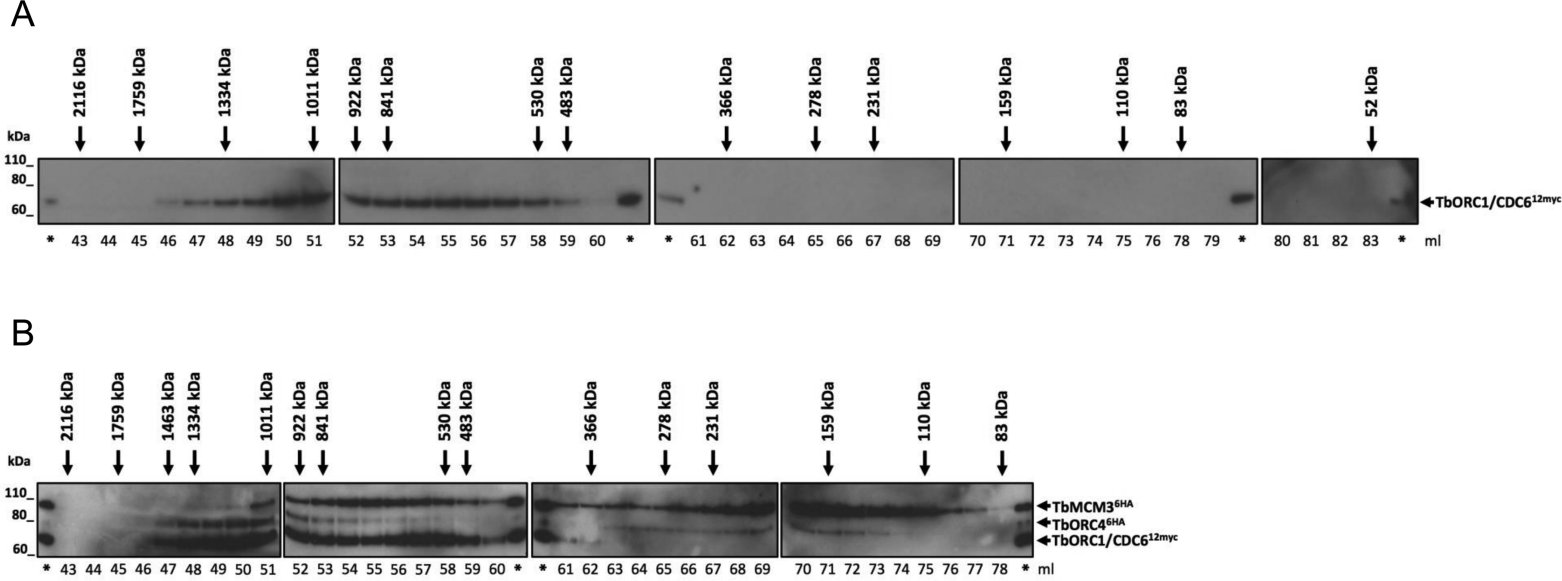
Gel filtration of *T. brucei* cell extracts. (**A**) Detection of TbORC1/CDC6–12myc with the myc antiserum in the different fractions resulting from gel filtration of TbORC1/CDC6 -/12myc cell line lysates. Multiple membranes spanning the fractions are aligned, and the estimated molecular weight is shown next to the corresponding fraction by a black arrow. The eluted volume (ml) corresponding to each fraction is depicted below each membrane. (*) pinpoints the lanes loaded with the lysed sample prior gel filtration, as a positive control in each western blot membrane. Size markers (kDa) are indicated. (**B**) Gell filtration of cell extracts from *T. brucei* PCF cells expressing either TbORC1/CDC6–12myc and TbORC4–6HA, or TbORC1/CDC6–12myc and TbMCM3–6HA. Extracts from the two cell lines were combined prior to gel filtration and proteins were detected with anti-myc and anti-HA antisera; expected sizes of the epitope tagged proteins are indicated.

Though each of TbORC4, Tb3120, Tb7980 and TbORC1B has been reported to interact with TbORC1/CDC6 (49,56), it remains unclear if these proteins interact together in a complex or individually with TbORC1/CDC6. To address this, gel filtration analysis was conducted. First, extracts from PCF TbORC1/CDC6–12myc expressing cells were subjected to size exclusion chromatography and the presence of TbORC1/CDC6–12myc in a wide range of collected fractions (from immediately after the void to the fraction potentially containing the monomeric form of TbORC1/CDC6) was analysed by western blot (Figure 8A). No signal was detected in fractions corresponding to monomeric TbORC1/CDC6–12myc (∼66 kDa), and instead TbORC1/CDC6–12myc was detected in fractions corresponding to proteins ranging from ∼1334 to 530 kDa, suggesting inclusion in a protein complex (Figure 8A). To examine the composition of the complex further, gel fitration was performed using PCF cells co-expressing TbORC1/CDC6–12myc and TbORC4–6HA, or TbORC1-CDC6–12myc and MCM3–6HA (Figure 8B). TbMCM3 has been previously described to interact with both TbORC1/CDC6 and TbORC1B (56), and its orthologue in *S. cerevisiae*, MCM3, has been shown to be the helicase subunit that mediates recruitment of MCM2–7 to DNA-bound ORC (81). Western blotting with both anti-myc and anti-HA antisera showed overlap of the fractions containing TbORC1/CDC6–12myc and both of the HA-tagged factors, corresponding to proteins ranging from ∼1011 to 530 kDa (Figure 8B). Unlike TbORC1/CDC6–12myc, both TbORC4–6HA and TbMCM3–6HA could be found in fractions corresponding to smaller proteins, though in neither case did these fractions match the expected size of the monomers (∼84 and ∼96 kDa, respectively). Whether these signals arise from some instability in association of TbORC4 and TbMCM3 with the larger complex or might represent inclusion of these factors in a subcomplex is unclear. Nonetheless, the overlapping signals of the three factors indicate that TbORC1/CDC6 and TbORC4 can interact in a large complex, which may additionally contain at least one subunit of the MCM2–7 helicase.

## DISCUSSION

This work has examined the machinery that acts in the initiation of nuclear DNA replication in *T. brucei*. Previously, only one factor, TbORC1/CDC6, had been shown to be involved in DNA replication, both through its localization to origins (55) and the demonstration that TbORC1/CDC6-targeting RNAi reduces the number of replicating cells in the population (54). Here we show that at least three TbORC1/CDC6-interacting factors, TbORC4, Tb3120 and TbORC1B (49,56), contribute to DNA replication, since RNAi of each of these factors impairs nuclear DNA replication and leads to similar growth and cell cycle defects. To date, interaction of TbORC1/CDC6 and these factors in an ORC-like complex has not been tested. We now provide evidence that TbORC1/CDC6 is stably associated with a high molecular weight complex, which also contains TbORC4 and perhaps TbMCM3. Taken together, these data are consistent with the initiation of DNA replication in *T. brucei* not being mediated by TbORC1/CDC6 alone, as previously suggested (53), but by a diverged ORC-like complex, which may be part of a pre-RC. Many of the proteins likely to constitute the putative *T. brucei* ORC are highly diverged in sequence (see below) from the canonical six ORC subunits found in yeast, mammals and *Drosophila*, each of which belongs to the opisthokont eukaryote supergroup (48). As a result, it remains possible that *T. brucei* ORC may not comprise the expected six subunits. In this regard, we show that TbORC1B is not a static component of *T. brucei* ORC, since its expression and/or nuclear localization is, uniquely among the *T. brucei* replication factors examined to date, limited to S phase. Thus, ORC architecture and regulation appear to be diverged features of replication initiation in *T. brucei*.

### Is TbORC1B a positive regulator of *T. brucei* nuclear DNA replication?

TbORC1B was initially identified as a second Orc1-like protein in *T. brucei* (56) and has been proposed to be a putative *T. brucei* ORC subunit (82,83). Super-resolution microscopy suggests that TbORC1B-12myc, TbORC1/CDC6–12myc and TbORC4–12myc all display related punctate localization throughout the nucleus. These findings may argue that TbORC1B is recruited to the putative ORC once in the nucleus. However, the same localization studies indicate that TbORC1B is not a static ORC subunit and may act instead as a positive regulator of *T. brucei* replication. Unlike the myc-tagged versions of TbORC1/CDC6, TbORC4, Tb7980 and Tb3120, TbORC1B-12myc is detectable in the nucleus of only ∼33% of the cells in an asynchronous population, being limited to S phase cells. A cell cycle transcriptome study (84) suggests that *TbORC1B* mRNA levels vary, peaking in late G1 and then reducing through S phase and G2 phases. Though these data may indicate mRNA turnover contributes to TbORC1B expression changes, the absence of detectable protein in early-mid G1 cells suggest that further, post-translational controls also operate. Although TbORC1B has been reported to interact with TbORC1/CDC6 during immunoprecipitation (56), reproducing this finding using a different combination of epitope tags has proved problematic (data not shown). Additionally, TbORC1B has not been recovered from IP-mass spectrometry analysis of TbORC1/CDC6–12myc or TbORC4–12myc, and the same analysis of TbORC1B-12myc does not recover any putative ORC component (data not shown). Taken together, these findings are consistent with the suggestion that any interaction between TbORC1B and the other ORC-like factors may be transitory or unstable, although it is possible that the limited expression of TbORC1B during the cell cycle makes detection of such interactions challenging.

The effects of TbORC1B expression silencing by inducible RNAi in PCF cells arise at least twice as quickly as those seen for TbORC1/CDC6 and TbORC4 (though are comparable in outcome). S phase limitation of TbORC1B expression most likely explains the rapidity and nature of the phenotypes seen after RNAi induction. At 6 h post-RNAi induction, no clear cell cycle perturbations are observed by DAPI staining, although flow cytometry analysis suggests a decrease in the number of G2/M phase (4n) cells. At the same time point, an ∼60% reduction in nuclear EdU incorporation is seen, suggesting that RNAi against TbORC1B impedes replication within a single PCF cell cycle (population doubling ∼12 h). In an asynchronous cell culture, the majority of *T. brucei* cells are in G1 (∼78%), when TbORC1B is not expressed. Thus, upon TbORC1B RNAi-induction, most cells will fail to express TbORC1B as they enter S phase, explaining why EdU incorporation is nearly undetectable 12 h after RNAi induction. The concomitant increase of cells that are morphologically 1N2K, but with unreplicated nuclei, is consistent with *T. brucei* kinetoplast and nuclear S phases being independent (73,85), indicating that loss of TbORC1B does not prevent the cells from continuing to replicate and segregate their mitochondrial genome. As RNAi induction progresses, both for TbORC1B and the other factors, the accumulation of 0N1K (zoid) cells is consistent with previous data (86), which showed that cytokinesis can occur in the absence of successful mitosis in PCF cells. Though the accumulation of aberrant cells, indicative of cell cycle alterations, is seen after RNAi of many essential factors, it is not a universal phenotypic response in these circumstances (65). The distinct cell cycle phenotypes seen after TbORC1B or TbORC1/CDC6 RNAi in BSF *T. brucei* cells, which are characterized not by the formation of zoids but frequently by multinucleate and multikinetoplast cells, seem most simply explained by the rapid activation of a cytokinesis cell cycle checkpoint in this life cycle stage.

The pattern of expression and localization of TbORC1B cannot be readily compared with any characterised pre-RC replication factor in other eukaryotes. Though TbORC1B was described as Orc1-like, the size and predicted domain composition of the polypeptide are perhaps more comparable with Cdc6 (87); indeed, protein BLAST searches with TbORC1B as query mainly recover Cdc6 sequences (data not shown). This may be telling, because Cdc6 expression and subcellular localization have been shown to be cell cycle regulated in many eukaryotes. Pre-RC assembly on origins, mediated by Cdc6, is conventionally considered to occur from late mitosis through G1. Upon entering S phase origins are activated and re-replication is prevented by some pre-RC disassembly, frequently by changes in Cdc6 localization or abundance. For instance, mammalian Cdc6 localizes to the nucleus in G1 phase and is transferred to the cytoplasm as the cell enters S phase (88,89). In *S. cerevisiae* and *S. pombe*, Cdc6 transcription is limited to M–G1 phases and the protein is degraded as the yeast cells enter S phase (90,91) via ubiquitylation and proteasome targeting (92–95). Thus, despite the potential sequence homology, TbORC1B's behaviour is quite unlike Cdc6. In addition, whether or not TbORC1B possesses ATPase activity, a central means by which Cdc6 exerts its activity is unclear (56). Regulation of the activity of ORC has also been reported in eukaryotes, including Orc1. In mammals, Orc1 has been shown to undergo selective degradation (96) and nuclear exclusion (97), but again these processes occur during S phase (98), and therefore differ from the S phase nuclear accumulation we see for TbORC1B. The closest analogy between TbORC1B expression dynamics and ORC in any other eukaryote is with ORC1 of *D. melanogaster*. In the fly, ORC1 is detectably expressed from late G1 phase through S, and is then degraded in M phase and during G1 by the Anaphase Promoting Complex through ubiquitin-mediated degradation guided by signals in the protein's N-terminus (99,100). However, mutation of *D. melanogaster* ORC1 to prevent M to G1 degradation has no effect on cell cycle progression, and it has been suggested that ORC1 expression dynamics may be needed for cell type-specific gene amplification by endoreplication(101,102). There is no evidence that *T. brucei* possesses any such discrete replication reactions. Furthermore, *D. melanogaster* ORC1 is highly conserved relative to opisthokont Orc1 subunits, including retention of an N-terminal BAH domain, and is part of a conventional six subunit ORC (22,35). As TbORC1B is more diverged in sequence from Orc1 than TbORC1/CDC6 and lacks any equivalent N-terminal sequence to that used in *D. melanogaster* ORC1 degradation, the analogy with the *D. melanogaster* factor, while intriguing, is limited. Instead, it appears likely that the S phase restriction of TbORC1B is central to the control of DNA replication in *T. brucei*.

TbORC1B nuclear localization dynamics are strikingly similar to *T. brucei* proliferating cell nuclear antigen (PCNA) (79), the sliding clamp involved in DNA polymerase association with DNA. In *T. brucei*, though not in related kinetoplastids (78,103,104), PCNA is only detectable in the nucleus of cells from late G1 phase to late S phase; indeed, TbPCNA nuclear localization shows similar large numbers of puncta as seen for TbORC1B, and similar partial overlap with newly replicated DNA (79). The available data are consistent with TbORC1B acting as a positive regulator of *T. brucei* nuclear DNA replication, since its dynamic presence in the nucleus correlates with the onset of DNA synthesis. If so, this would be a highly unorthodox strategy for replication control, and how TbORC1B might exert such a function is unclear. In other eukaryotes, interaction between ORC and the MCM helicase is mediated by Cdt1 (2,16), a factor that bears no sequence comparison with TbORC1B. Nonetheless, TbORC1B, like TbORC1/CDC6, has been reported to interact with the TbMCM helicase subunit TbMCM3 (56). In this regard, the potential that *T. brucei* ORC and MCM interact rather stably (see below) might indicate that TbORC1B acts in an unprecedented way to influence the dynamics of pre-RC formation or regulation in the parasite.

### What is the composition of *T. brucei* ORC?

**Figure 9.**
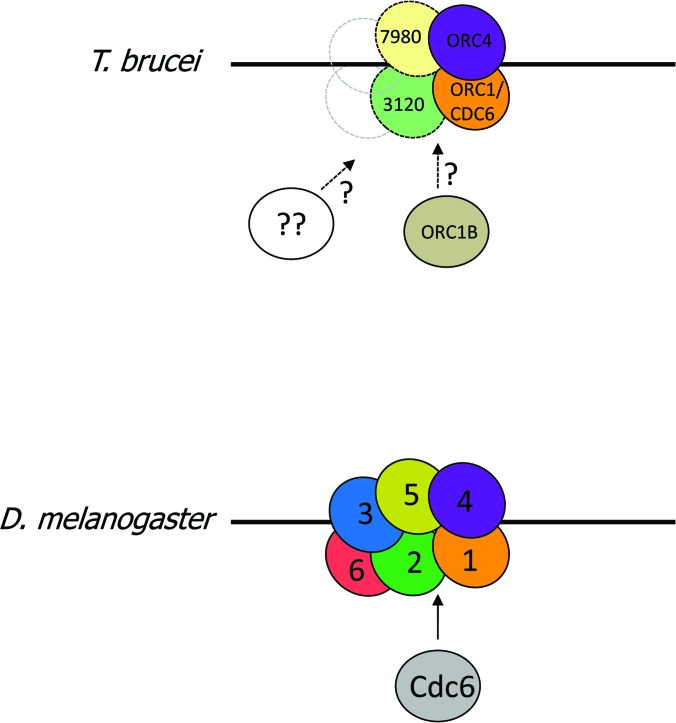
Potential ORC architecture in *T. brucei*. Architecture of the *D. melanogaster* origin recognition complex (ORC; composed of Orc subunits numbered 1–6), which interacts (arrow) with the Orc1-related factor Cdc6, is shown based on the structure determined by (22). In *T. brucei*, recognisable ORC subunit orthologues of Orc1 and Orc4 (TbORC1/CDC6 and TbORC4, respectively) are identified using the same colours and solid outlines, while putative orthologues of Orc2 and Orc5 (Tb3120 and Tb7980, respectively) are shown by dotted circles and lighter colours; subunits that are absent or highly diverged are shown by unfilled dotted circles. TbORC1B interacts with TbORC1/CDC6, but appears to not be a static ORC component, and hence its inclusion in ORC is uncertain.

Gel filtration analysis shown here indicates that TbORC1/CDC6 is present, with TbORC4, in a high molecular weight complex in PCF cells, providing the first indication that an ORC-like complex is present in *T. brucei*. However, identifying the remaining constituent proteins of such an ORC has been challenging and therefore its composition remains to be fully established (Figure 9). To date, we can place one other factor, along with TbORC1/CDC6 and TbORC4, in *T. brucei* ORC with reasonable confidence: Tb3120. RNAi of each of these three factors results in impaired DNA replication, in each case leading to related cell cycle defects in PCF cells. Moreover, each of the three proteins displays similar punctate nuclear localization throughout the cell cycle, and each has sequence characteristics of ATPase function (see below). We suggest that it is likely that Tb7980 is also an ORC component: though we could not recover stem-loop RNAi cells to test for loss of EdU incorporation after knockdown, in previous analysis loss of the factor resulted in the same cell cycle defects seen after TbORC1/CDC6, TbORC4 or Tb3120 RNAi (49), which were shown here to follow from impaired replication. In addition, Tb7980 has homology to AAA+ ATPases, a common feature of ORC subunits (see below), and shows the same constitutive nuclear localization as the three other *T. brucei* ORC candidates.

A complexity in understanding *T. brucei* ORC is the size of the putative complex in which TbORC1/CDC6 is found (greater than 530 kDa and perhaps as large as 1334 kDa, from gel filtration): this is too large to be composed of only TbORC1/CDC6, TbORC4, Tb7980 and Tb3120, which would be ∼305 kDa (assuming each protein interacts in single copy). It seems unlikely that TbORC1B interacts stably with the putative ORC, but even if it did, the size of the predicted complex would only be ∼371 kDa. One explanation for this dichotomy is that the TbORC1/CDC6 immunoprecipitations conducted to date (49,56) have failed to recover further *T. brucei* ORC components that are present and have escaped sequence-based identification (Figure 9). However, perhaps the most likely explanation for the large size of the TbORC1/CDC6-containing complex is revealed by the demonstration at least some of TbMCM3 and TbORC4 co-elute with TbORC1/CDC6. These data suggest the complex identified by gel filtration could be the *T. brucei* pre-RC, composed of ORC bound to the *T. brucei* MCM2–7 helicase, which would amount to ∼857 or ∼923 kDa (without or with TbORC1B, respectively). However, this size estimate is consistent only with the presence of a single copy of the TbMCM2–7 hexamer, whereas *in vitro* studies have shown that dimers of MCM2–7 associate with ORC in the pre-RC (105). Thus, whether the complex we detect represents a ‘true’ pre-RC awaits further analysis.

### Why might ORC in kinetoplastids be diverged?

The organisation of the six core subunits (Orcs1–6) of ORC, and their association with Cdc6, MCM2–7 and Cdt1 in the pre-RC, has been revealed by EM and crystallographic studies of the complexes from *S. cerevisiae* (11,32–34) and *D. melanogaster* (22,35). However, it remains unclear what role is played by each ORC subunit, in particular why six stably interacting factors are needed to mediate DNA interaction and pre-RC formation. In fact, it is increasingly clear that the canonical six-subunit ORC, as detailed in the opisthokonts, is less well conserved in other eukaryotic groupings, with several of the Orc subunits being undetectable through sequence homology (48,49). Irrespective of whether these data indicate that some ORC components have been dispensed with, have substantially diverged in sequence, or have even been supplanted by other factors, variant ORC architecture provides insight into core ORC functions and lineage-specific adaptations. The data we present probes ORC architecture in *T. brucei*, an eukaryote belonging to the excavata supergroup, which may have diverged from the opisthokonts ∼1 billion years ago (106). From these data, what ORC features are conserved and diverged, and what might these differences reveal? Assuming a *T. brucei* ORC structure comparable with *D. melanogaster* (22), TbORC1/CDC6 and TbORC4 represent the conserved core of ORC (Figure 9), consistent with the *Drosophila* orthologues of these factors being adjacent in the complex and evidence for functional interaction through shared ATPase motifs (22,107). In this regard, it is notable that TbORC1/CDC6 and TbORC4 are the only two *T. brucei* ORC factors that display clear, primary sequence-derived orthology with characterised ORC subunits in other eukaryotes (49), albeit with TbORC1/CDC6 (and orthologues in other kinetoplastids) lacking an N-terminal BAH domain, which is conserved in all other studied eukaryotes. In the light of the recently solved structure of *D. melanogaster* ORC (22), we tentatively propose that Tb3120 is related to Orc2: though eukaryotic ORC factors are not recovered in BLASTp searches using Tb3120 as a query, structural modelling predicts that *D. melanogaster* Orc2 and Tb3120 are related in their C-termini, a region including the Orc2 WH domain (Supplementary Figure S11A and B). In addition, though there is no evidence for an intact AAA+ ATPase domain in Tb3120, non-canonical Walker A and Walker B motifs, which possess sequence patterns characteristic of Orc2 proteins (33), can be identified (Supplementary Figure S11C). Indeed, these motifs lie within two regions of Tb3120, separated by 258 aa, that display weak evidence of Orc2 domain homology in Pfam searches (Supplementary Figure S11A). Nonetheless, Tb3120 (as well as its *T. cruzi* and *L. major* counterparts) remains strikingly large compared with other Orc2 subunits (1018 residues, compared with ∼580–620 in opisthokonts), most likely because the kinetoplastid proteins have evolved an N-terminal extension and an intra-AAA+ ATPase insertion (Supplementary Figure S11A), for reasons that have not been tested. Primary sequence homology searches reveal no ORC subunit orthology for Tb7980 (49), but do reveal AAA+ ATPase homology at the N-terminus (Supplementary Figure S12A). Structural modelling now suggests extended homology between Tb7980 and *D. melanogaster* Orc5 (Supplementary Figure S12A and B), including alignment of the Walker A and Walker B motifs (Supplementary Figure S12C). This tentative identity for Tb7980 as Orc5 would be consistent with the order of subunit interactions in *D. melanogaster* ORC (22), placing Tb7980 adjacent to TbORC4 in the *T. brucei* ORC (Figure 9). If this proposed *T. brucei* ORC-like organisation is accurate, it suggests a relatively well-conserved Orc1-Orc4 core, with increasing divergence as the complex extends to Orc5 (Tb7980) and Orc2 (Tb3120). Whether *T. brucei* ORC possesses six subunits, with even greater subunit divergence beyond Orc1–4–5–2, or whether subunits equivalent to Orc3 and Orc6 are absent in *T. brucei*, is currently unknown. Nonetheless, this topological organization reflects eukaryote-wide predictions of ORC presence and absence (48,49) and concurs with some predictions of ORC composition in the last common eukaryotic ancestor (48).

ORC provides at least two functions in DNA replication: interaction with origins and recruitment of the MCM helicase in the pre-RC. Either of these functions could explain the divergence of *T. brucei* ORC. DNA binding in eukayotic ORCs displays considerable variation, with sequence specific binding by *S. cerevisiae* ORC (18) and apparently sequence-independent DNA interactions by ORC in *D. melanogaster* and mammals (108,109). Though to some extent diverged from archaeal Orc1/Cdc6 DNA interaction (23), the crystal structure of *D. melanogaster* ORC suggests that the WH domains that are common to Orcs1–5 contribute to DNA binding (22). In *T. brucei*, TbORC1/CDC6 binding sites localize to the boundaries of the RNA Pol II multigenic transcription units in the core genome, with a preference for the transcription start sites but without detectable sequence specificity (55). Sequence features of typical eukaryotic RNA pol II promoters are lacking in the parasite genome, and transcription initiation may rely on lineage-specific chromatin cues (110). In this light, the absence of a BAH domain in TbORC1/CDC6, or in any presently identified interacting partner, is striking, since this feature contributes to origin selection in opisthokonts (111,112). More broadly, the variant architecture of kinetoplastid ORC may also reflect lineage-specific adaptations in how the complex interacts with the genome, perhaps due to binding to variant transcription machinery or chromatin. Indeed, variation in this key step of origin designation may be widespread, since the ORC of *T. thermophila* can recruit an rRNA species for DNA binding (113,114). Structural data suggest that ORC in both *S. cerevisiae* and *D. melanogaster* undergoes substantial reorganisation on binding Cdc6 and through ATP hydrolysis, which allows recruitment of Cdt1-MCM2–7, again potentially through ORC WH domains (11,22,32). To what extent such changes might be altered in *T. brucei* ORC if some subunits are absent or have been replaced by factors lacking AAA+ ATPase domains (thereby limiting oligomerization or inter-subunit motif interactions, which are typical of AAA+ ATPases) (4) is hard to evaluate in the absence of structural data. However, it may indicate a change in the nature or dynamics of *T. brucei* ORC interaction with the replicative helicase, perhaps consistent with the observation that at least one subunit of what appears to be a typical MCM2–7 hexamer (49,56) may interact in a complex with TbORC1/CDC6 and TbORC4. Indeed, the diverged ORC architecture might be related to how the variant, positive acting role of TbORC1B influences pre-RC assembly or regulation during *T. brucei* nuclear DNA replication.

## Supplementary Material

Supplementary Data

SUPPLEMENTARY DATA
