# Supplementary material for: Diverged composition and regulation of the Trypanosoma brucei origin recognition complex that mediates DNA replication initiation
Source: Nucleic Acids Res. 2016 Mar 6;44(10):4763–84. doi: 10.1093/nar/gkw147 (PMC4889932; doi:10.1093/nar/gkw147)
Supplement: Supplementary Data [file gkw147_Supplementary_Data.zip › nar-03225-v-2015-File011.pdf]

Diverged composition and regulation of the *Trypanosoma brucei* origin recognition complex that mediates DNA replication initiation

Catarina A. Marques, Calvin Tiengwe, Leandro Lemgruber, Jeziel Damasceno, Alan Scott, Daniel Paape, Lucio Marcello and Richard McCulloch

Supplementary figure legends

Fig.S1. Representative images of cells categorised as 'other' in Figures 1 and 2. Images correspond to the ORC1/CDC6 RNAi cell line, 96h after RNAi induction by tetracycline. The cells have a variable configuration of nucleus and kinetoplast numbers, and no predominant nucleus to kinetoplast ratio was observed. In addition, all cells present an irregular cellular shape. Scale bar represents 5 µm.

Fig.S2. FACS profiles following RNAi against TbORC1/CDC6, TbORC4 and Tb3120 in procyclic form *T. brucei*. Histograms representing the distribution of cell populations according to DNA content, after staining with propidium iodide and analysis by flow cytometry, is shown. Approximately 30,000 cells were analysed per sample. Histograms from RNAi induced cells (Tet+, red line) and from uninduced control cells (Tet-, black line) are shown for various time points, which relate to emergence of aberrant cell types as described in Fig.1

Fig. S3. Analysis of the growth in cell culture, DNA replication efficiency and cell cycle progression of PCF *T. brucei* cells expressing endogenously 12myc tagged replication factors. A. Confirmation of the expression of TbORC1/CDC6, TbORC1B, TbORC4, Tb7980 and Tb3120 tagged with 12myc by western blot of whole cell extracts. **B.** Growth curves are shown of untagged *T. brucei* PCF cells (927 wt) relative to TbORC1/CDC6 -/12myc, TbORC1B 12myc, TbORC4 12myc, Tb3120 12myc and Tb7980 -/12myc cells. In each case the different cells were inoculated at a concentration of 1 x 10^6 cells/ml and cell density was then assessed every 24 h. Values represent the the mean of two independent experiments, and the error bars show the SEM. **C.** Cells were stained with DAPI, and the different cell cycle stages quantified based on N-K configuration. A minimum of 125 cells was counted per experiment, and the percentage of cells in each cell cycle stage was calculated from the total number of cells counted per cell line. The mean of two (Tb3120 and Tb7980) or three (TbORC1/CDC6, TbORC1B and TbORC4) independent experiments is shown and error bars represent the SEM. **D.** Cells at a density of 1 x 10^7 were collected and incubated with EdU for 3 h, and then stained for EdU detection and quantification by fluorescent microscopy. A minimum of 125 cells was counted per experiment, and the percentage of cells showing nuclear EdU signal was calculated from the

total number of cells counted per cell line. Data shown represent the mean % of EdU positive cells from two independent experiments, and the error bars show the SEM.

Fig.S4. Quantification of putative ORC factor subcellular localisation through the cell cycle. PCF *T. brucei* cells expressing TbORC4-12myc (**A**), Tb3120-12myc (**B**), or 12myc-Tb7980 (**C**) were fixed and stained with DAPI or with AlexaFluor® 488-conjugated anti-myc antibody. Intensity of the DAPI and myc signals is represented (dots) as the mean pixel intensity within a circular region of interest (21 x 21 pixels), drawn around each individual cell nucleus; for the myc data, the red dotted line represents the average background signal measured in 927wt cells that do not express any tagged protein. In each case, cells are separated by cell cycle stage, determined by N-K ratio in the DAPI images: 1N1K cells (G1 phase), 1N1eK cells (S phase), 1N2K cells (G2/M phase), and 2N2K cells (post-mitosis). In all cases, >240 cells were analysed; median values are represented, with error bars depicting the interquartile range. Statistical significance between the different cell cycle stages was assessed using the Kruskal-Wallis non-parametric test: (*) p-value < 0.05; (**) p-value < 0.01; (***) p-value < 0.001; (****) p-value <0.0001.

Fig.S5. Super resolution imaging of TbORC1/CDC6 through the cell cycle. PCF *T. brucei* cells expressing TbORC1/CDC6-12myc were incubated for 3 h with 150 µm EdU, fixed and stained with DAPI and with AlexaFluor® 488-conjugated anti-myc antibody, while EdU was detected with AlexaFluor® 594-conjugated azide. Images were acquired with a Zeiss Elyra super-resolution microscope system in SIM mode. In each case, representative maximum projection images are shown of cells in the different cell cycle stages, determined by N-K ratio in the DAPI images: 1N1K cells (G1 phase), 1N1eK cells (S phase), 1N2K cells (G2/M phase), and 2N2K cells (post-mitosis). In each case, the rightmost image shows a merge of the EdU (red), anti-myc antiserum (green) and DAPI (blue) signals, while the three images from left to right show the individual EdU, anti-myc antiserum and DAPI signals in greyscale. The three signal intensities for each cell type are quantified in the plots to the left; signal intensities (y-axes, arbitrary units) were analysed in a horizontal line across the boxed area surrounding the nucleus.

Fig.S6. Super resolution imaging of TbORC4 through the cell cycle. This figure is organized as in Fig.S5 and cells were prepared in the same way, except that *T. brucei* PCF cells expressing TbORC4-12myc were used.

Fig.S7. Super resolution imaging of TbORC1B through the cell cycle. This figure is organized as in Fig.S5 and cells were prepared in the same way, except that *T. brucei* PCF cells expressing TbORC1B-12myc were used.

Fig.S8. Super resolution imaging of TbMCM3 through the cell cycle. This figure is organized as in Fig.S5 and cells were prepared in the same way, except that *T. brucei* PCF cells expressing TbMCM3-12myc were used.

Fig.S9. Cell-cycle dependent nuclear localisation of TbORC1/CDC6, TbORC4, Tb3120, TbORC1B and Tb7980 in bloodsteam form *T. brucei*. Immunofluorescent detection of TbORC1/CDC6 -12myc (**A**), TbORC4

-12myc (**B**), Tb3120-12myc (**C**), TbORC1B-12myc (**D**) or 12myc-Tb7980 (**E**) with anti-myc antiserum (middle row) in 1N1K cells (G1 phase), 1N1eK cells (S phase), 1N2K cells (G2/M phase), and 2N2K cells (post-mitosis); the same cells are shown stained with DAPI (top row), and the cell outline is shown by DIC (lower row). Images were acquired using a DeltaVision imaging system and deconvolved using the ratio conservative method, on SoftWoRx software. The Scale bar represents 5 µm.

Fig.S10. Cell-cycle dependent nuclear localisation of TbORC1B in BSF *T. brucei*.A. Confirmation of the expression of TbORC1/CDC6, TbORC1B, TbORC4, Tb7980 and Tb3120 tagged with 12myc by western blot of whole cell extracts. **B.** Percentage of cells containing nuclear myc signal in untagged *T. brucei* BSF cells (427 wt) relative to the cells expressing TbORC1/CDC6-12myc, TbORC1B-12myc, TbORC4-12myc, 12myc-Tb7980 or Tb3120-12myc, each from the endogenous locus; the mean of two independent experiments is shown (>125 cells each), and error bars show SEM. **C.** The proportion of cell cycle stages displaying TbORC1B-12myc signal is shown either as the percentage of total cells (insert), or as the percentage of positive cells (main graph), as determined by N-K ratio (dark blue, 1N1K cells; light blue, 1N1eK cells; yellow, 1N2K cells, red, 2N2K). The mean is shown from two independent experiments, and the error bars depict standard deviation. **D.** Percentage of individual cell cycle stage cells that display TbORC1B-12myc signal; the mean of two independent experiments is represented and error bars show standard deviation. Statistical significance between the different groups was assessed using the one-way ANOVA parametric test: (**) p-value < 0.01; (***) p-value <0.001. Note that only one single 2N2K cell was found to have signal, and thus the resulting mean is statistically different from the other three groups (p-value not represented).

Fig.S11. Tb3120 may be a distant orthologue of eukaryotic Orc2. A. A schematic representation of predicted AAA+ and Winged helix (WHD) domains domains and Walker A and B motifs in the polypetide sequences of *D. melanogaster* Orc2 (DmOrc2) and Tb3120; sizes are indicated (number of amino acids, aa) and domains are coloured as shown. Note that domains highlighted with (*) indicates that primary sequence predictions of these domains (from Pfam) are insignificant matches. **B.** Raptor X structural modeling of Tb3120 (p-value 2.77 e-03) based on the solved structure of DmOrc2 (Bleichert et al, 2015); the region modellled is indicated in **A** by a green dashed line. **C.** Alignment of predicted Walker A and Walker B motifs in Tb3120 (and orthologues in *T. cruzi* and *L. major* : Tc3120 and Lm3120) and Orc2 subunits of model eukaryotes; locations and sequences of the motifs in Tb3120 and DmOrc2 are highlighted in **A**.

Fig.S12. Tb7980 may be a distant orthologue of eukaryotic Orc5. A. A schematic representation of predicted AAA+ and Winged helix (WHD) domains domains and Walker A and B motifs in the polypetide sequences of *D. melanogaster* Orc5 (DmOrc5) and Tb7980; sizes are indicated (number of amino acids, aa) and domains are coloured as shown . Note that domains highlighted with (*) indicates that primary sequence predictions of these domains (from Pfam) are insignificant matches. **B.** Raptor X structural

modeling of Tb7980 (p-value 1.5 e-05) based on the solved structure of DmOrc5 (Bleichert et al, 2015); the regions modellled are indicated in **A** (green dashed line). **C.** Alignment of predicted Walker A and Walker B motifs in Tb7980 (and orthologues in *T. cruzi* and *L. major* : Tc7980 and Lm7980) and Orc5 subunits of model eukaryotes; locations and sequences of the motifs in Tb7980 and DmOrc5 are highlighted in **A**.

References

Bleichert F, Botchan MR, Berger JM (2015) Crystal structure of the eukaryotic origin recognition complex. *Nature* **519:** 321-326

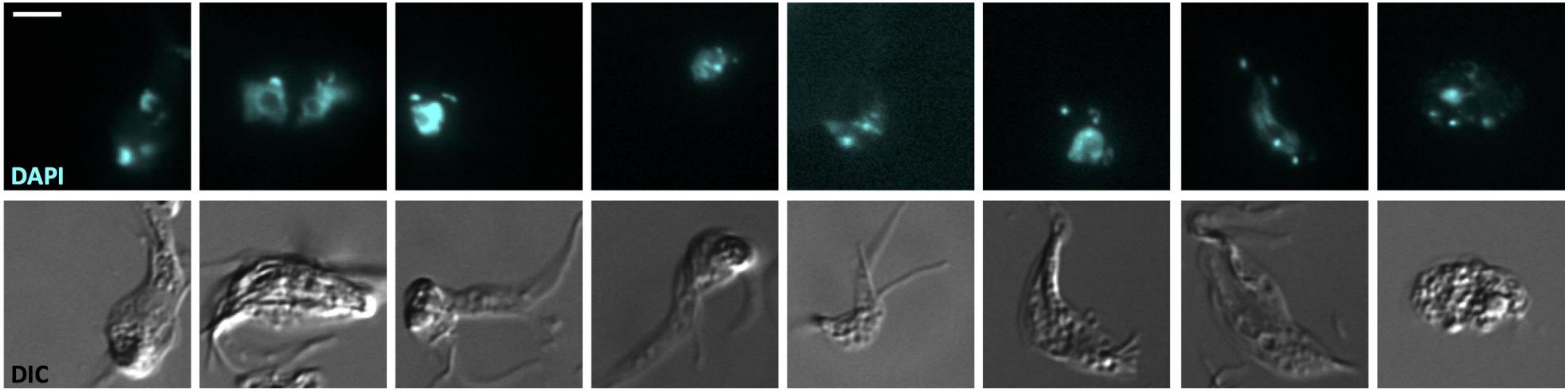

Fig.S1

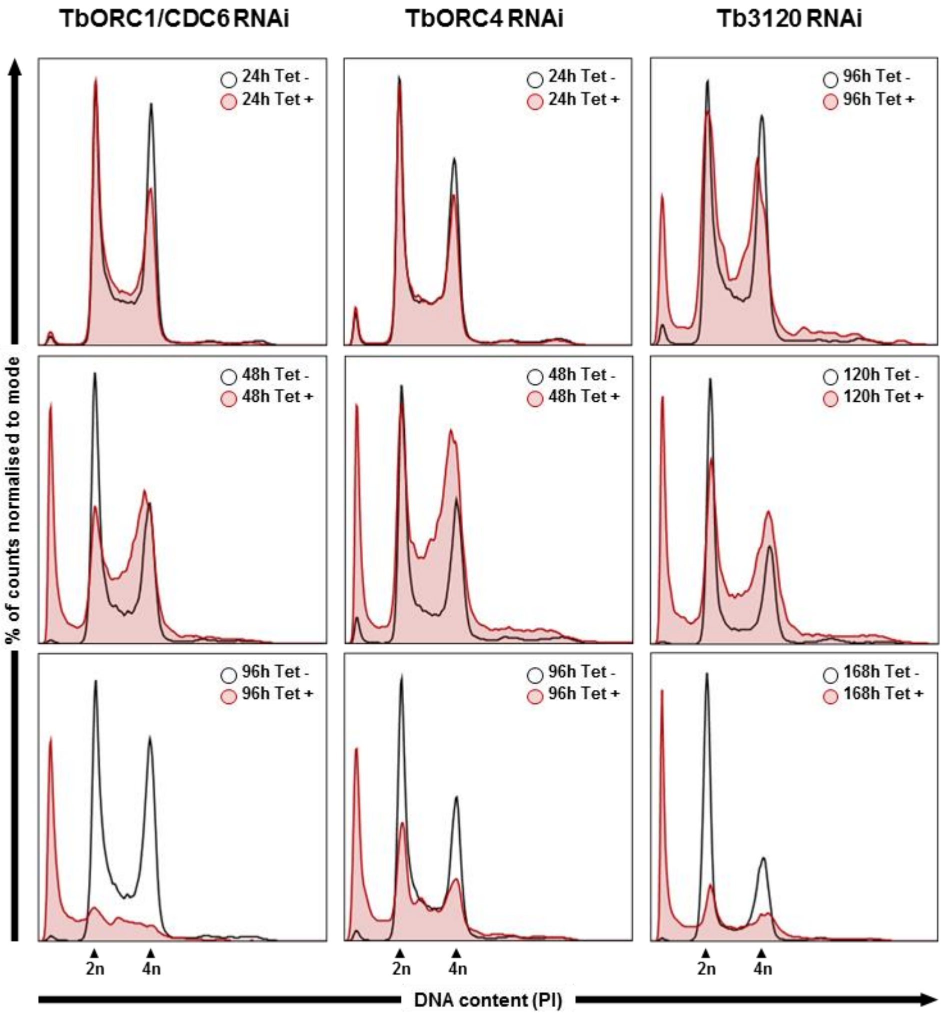

TbORC1/CDC6 RNAi
TbORC4 RNAi
Tb3120 RNAi
24h Tet -
24h Tet +
24h Tet -
24h Tet +
96h Tet -
96h Tet +
48h Tet -
48h Tet +
48h Tet -
48h Tet +
120h Tet -
120h Tet +
96h Tet -
96h Tet +
96h Tet -
96h Tet +
168h Tet -
168h Tet +
% of counts normalised to mode
2n
4n
2n
4n
2n
4n
DNA content (PI)

Fig.S2

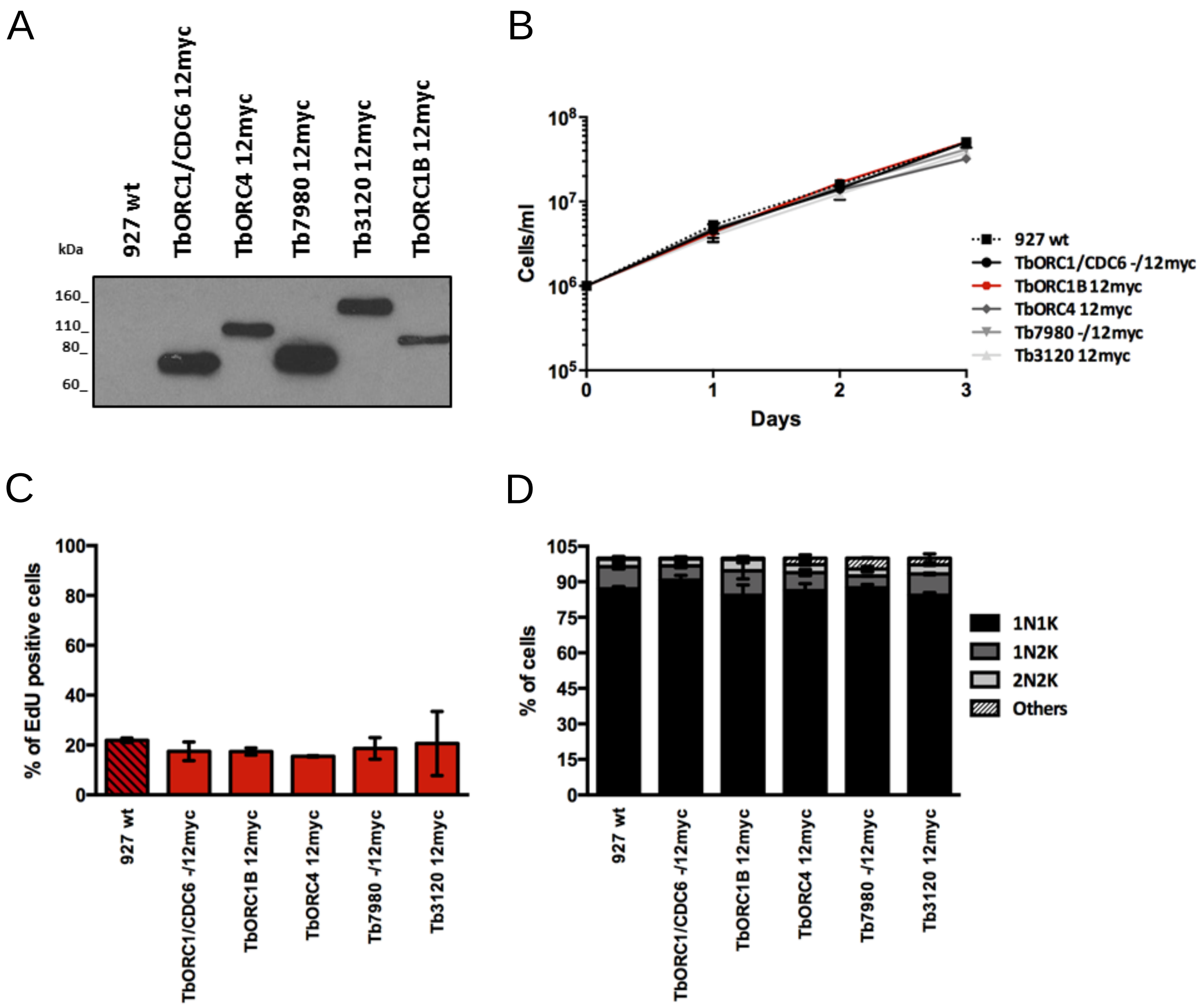
A
927 wt
TbORC1/CDC6 12myc
TbORC4 12myc
Tb7980 12myc
Tb3120 12myc
TbORC1B 12myc
kDa
160
110
80
60
B
Cells/ml
Days
927 wt
TbORC1/CDC6 -/12myc
TbORC1B 12myc
TbORC4 12myc
Tb7980 -/12myc
Tb3120 12myc
C
% of EdU positive cells
927 wt
TbORC1/CDC6 -/12myc
TbORC1B 12myc
TbORC4 12myc
Tb7980 -/12myc
Tb3120 12myc
D
% of cells
1N1K
1N2K
2N2K
Others
927 wt
TbORC1/CDC6 -/12myc
TbORC1B 12myc
TbORC4 12myc
Tb7980 -/12myc
Tb3120 12myc

Fig.S3

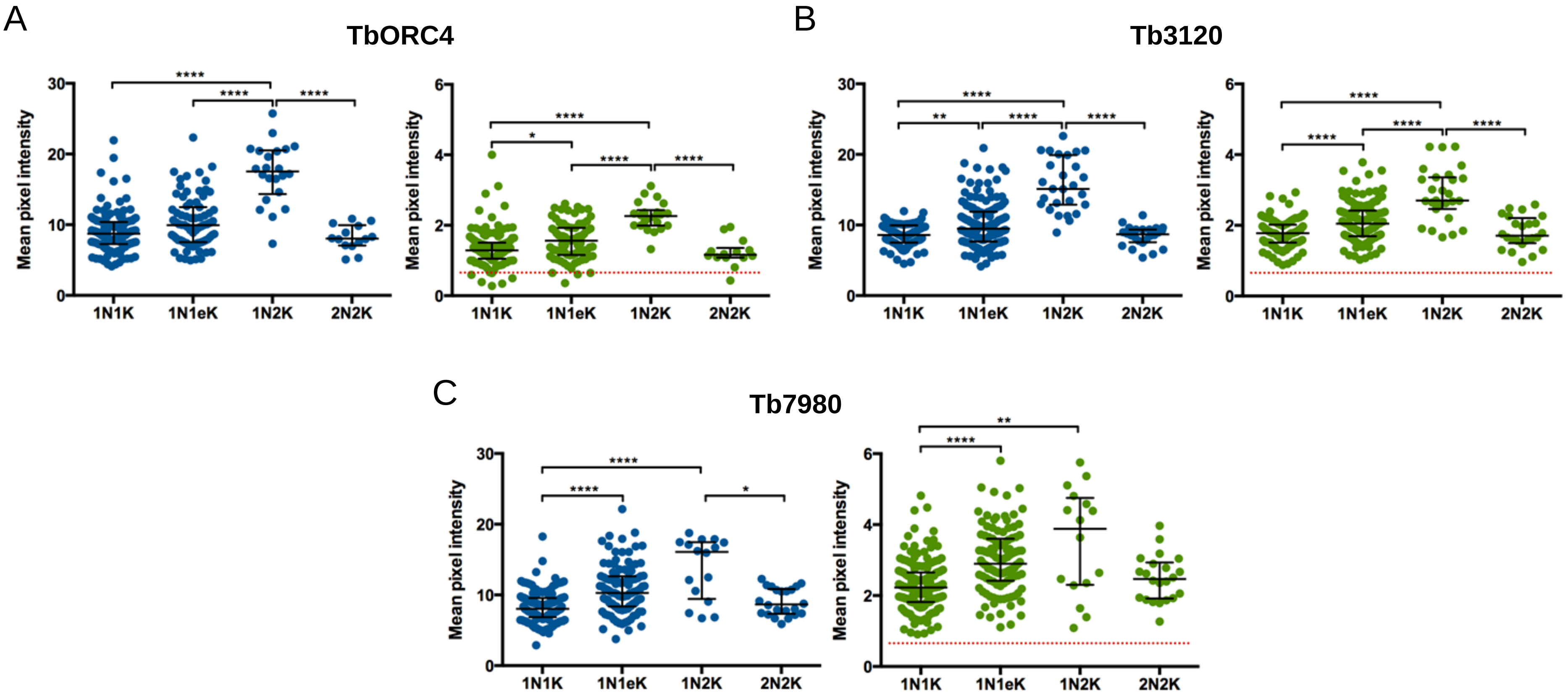
A
TbORC4
B
Tb3120
C
Tb7980
Mean pixel intensity
0
10
20
30
2
4
6
1N1K
1N1eK
1N2K
2N2K
****
**
*

Fig.S4

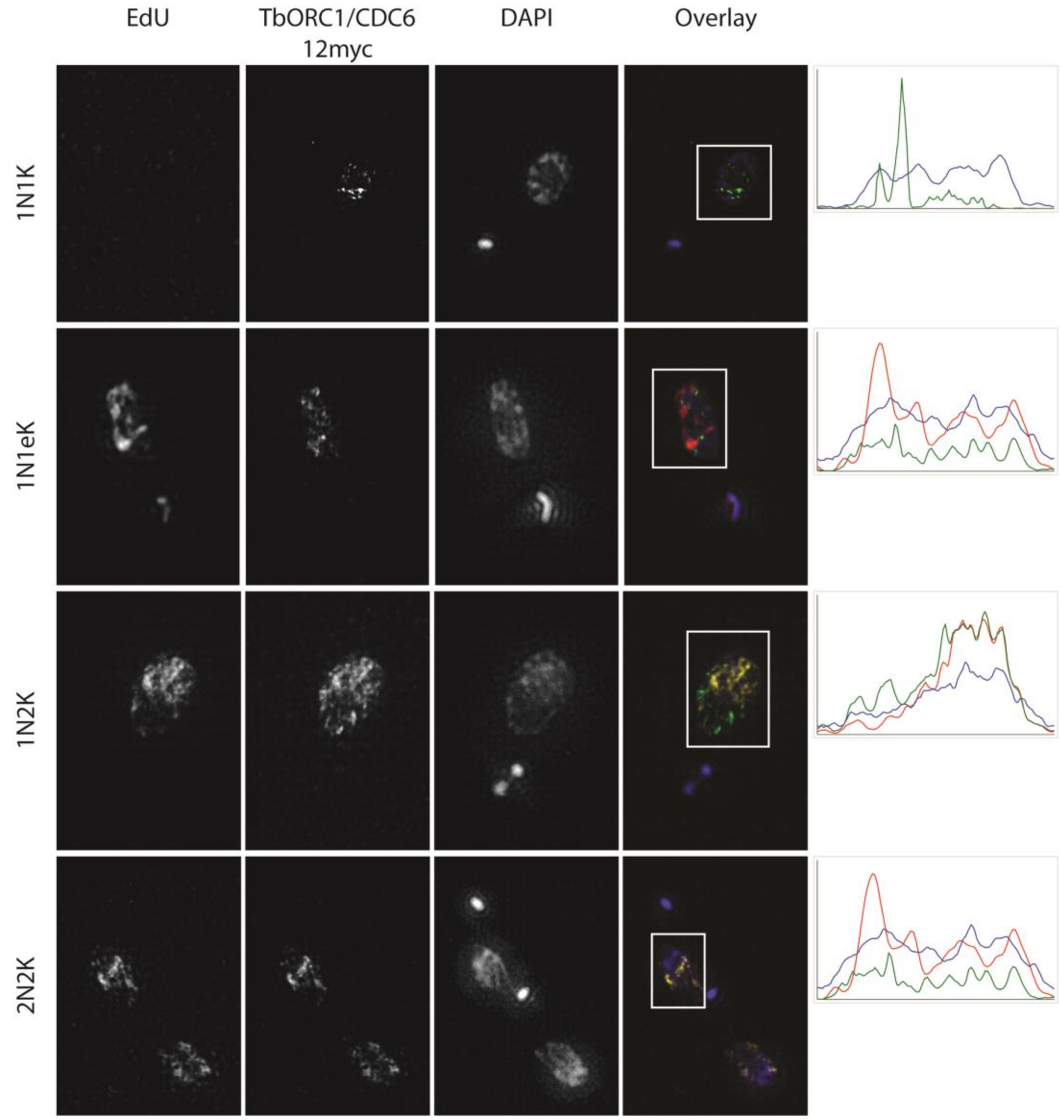

Fig.S5

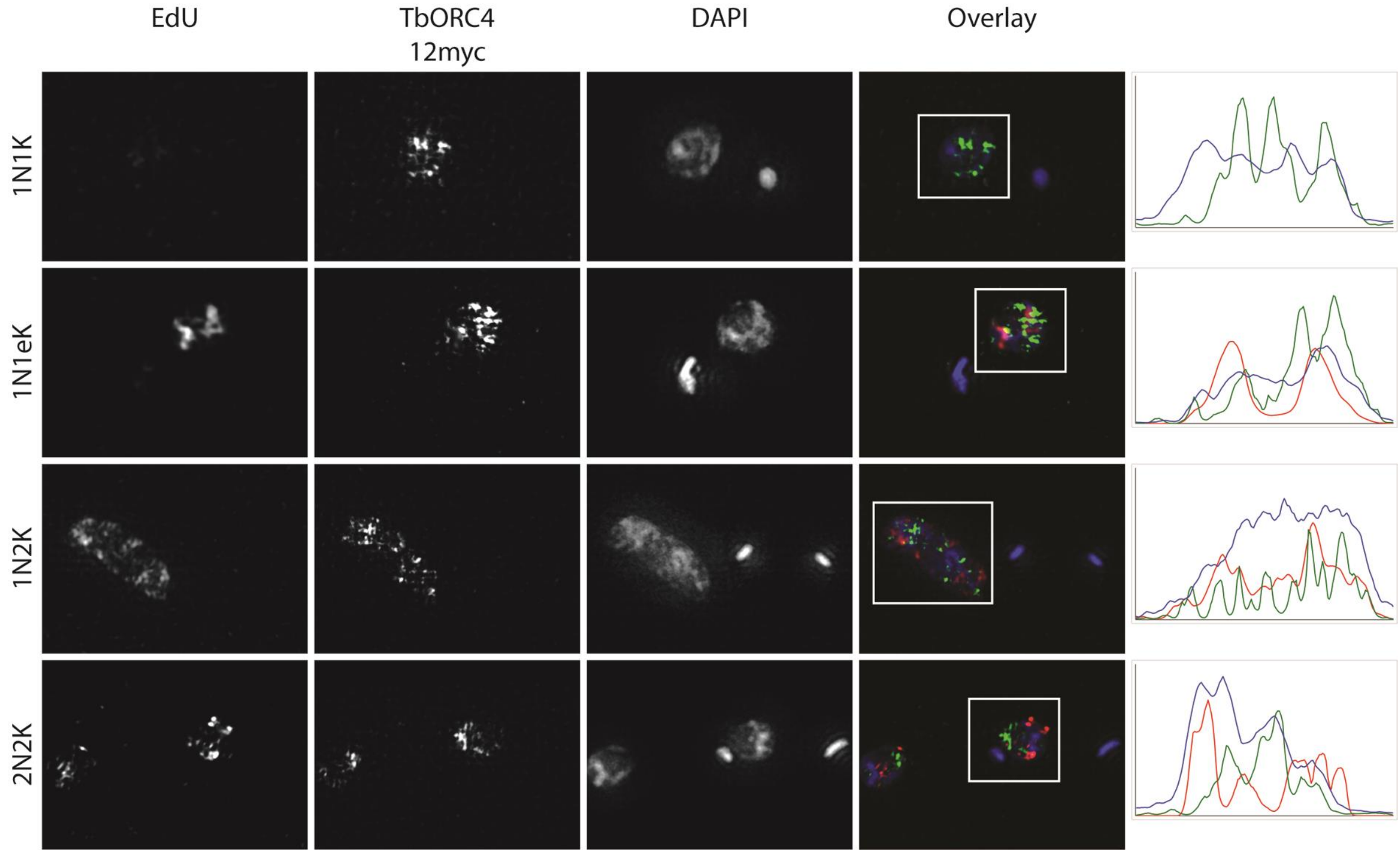

Fig.S6

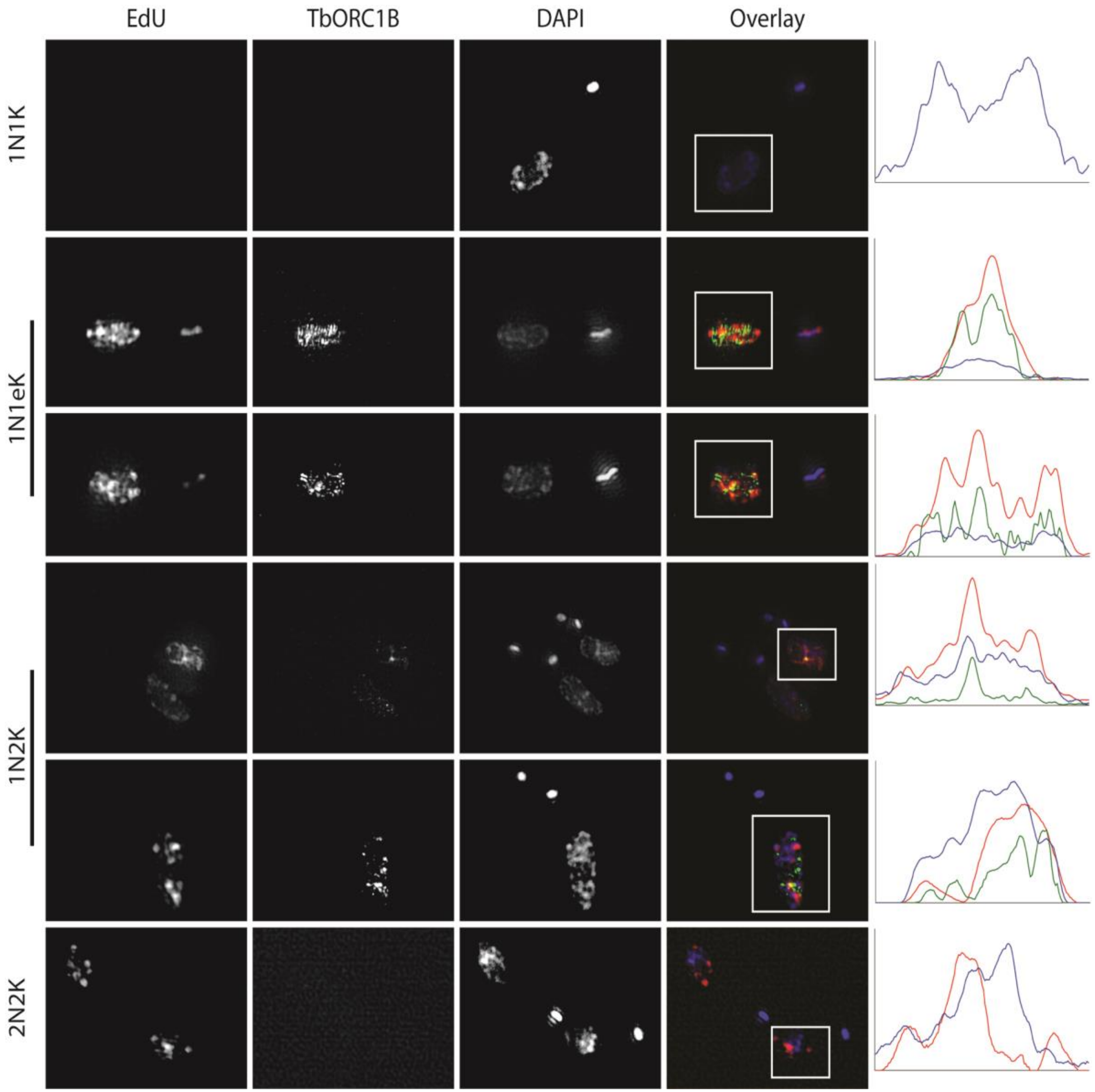

Fig.S7

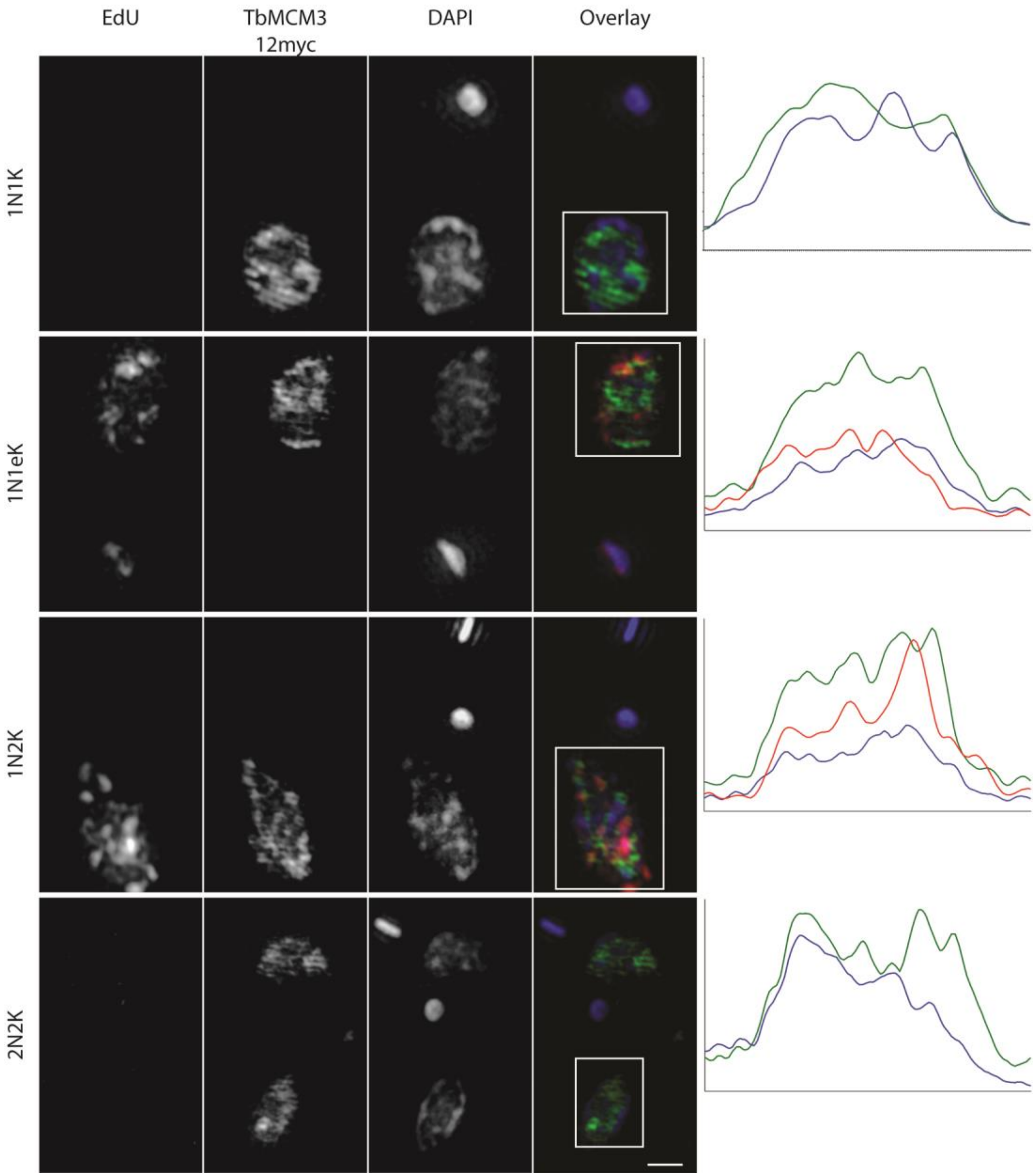

Fig.S8

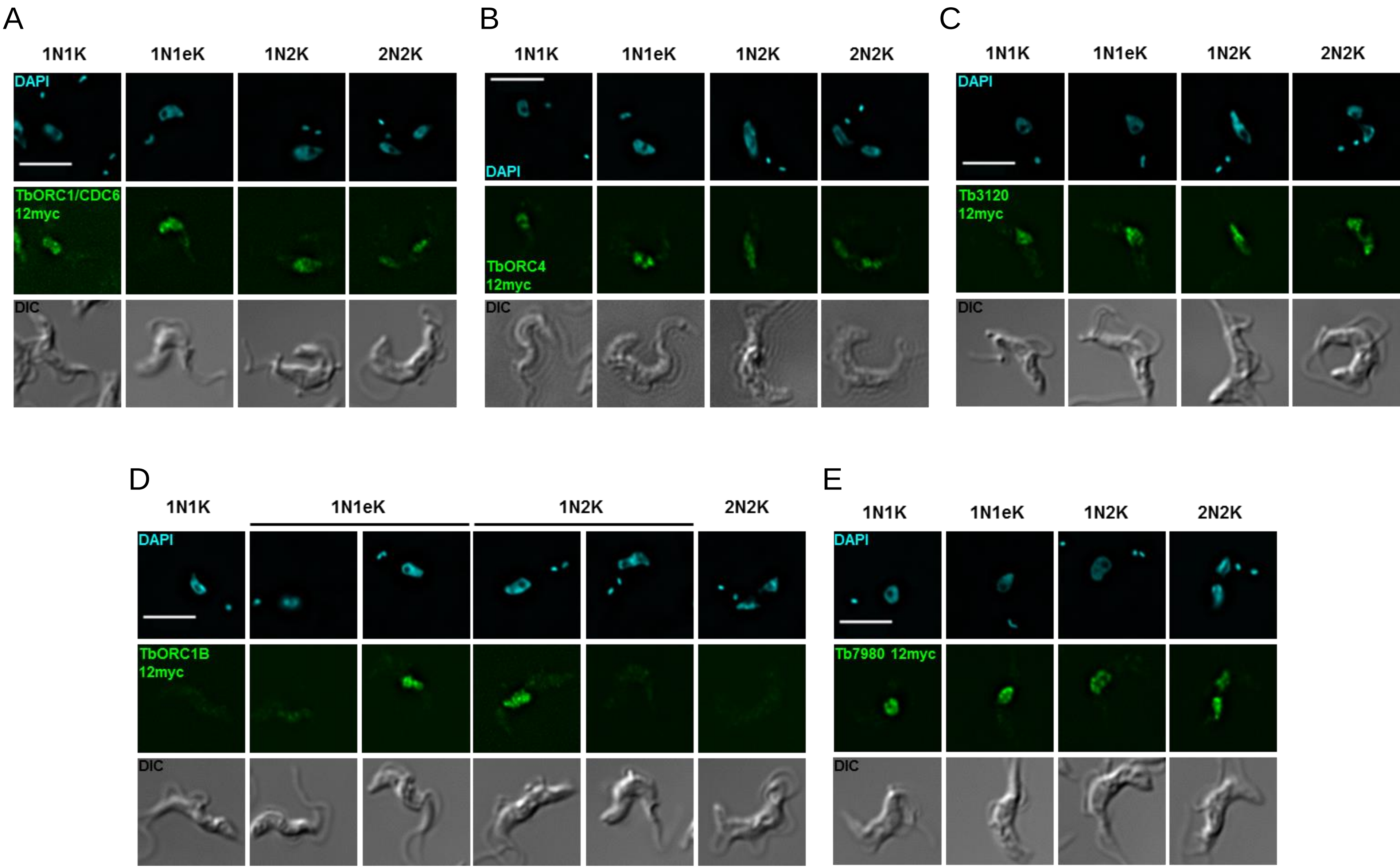
A
1N1K
1N1eK
1N2K
2N2K
DAPI
TbORC1/CDC6
12myc
DIC
B
1N1K
1N1eK
1N2K
2N2K
DAPI
TbORC4
12myc
DIC
C
1N1K
1N1eK
1N2K
2N2K
DAPI
Tb3120
12myc
DIC
D
1N1K
1N1eK
1N2K
2N2K
DAPI
TbORC1B
12myc
DIC
E
1N1K
1N1eK
1N2K
2N2K
DAPI
Tb7980 12myc
DIC

Fig.S9

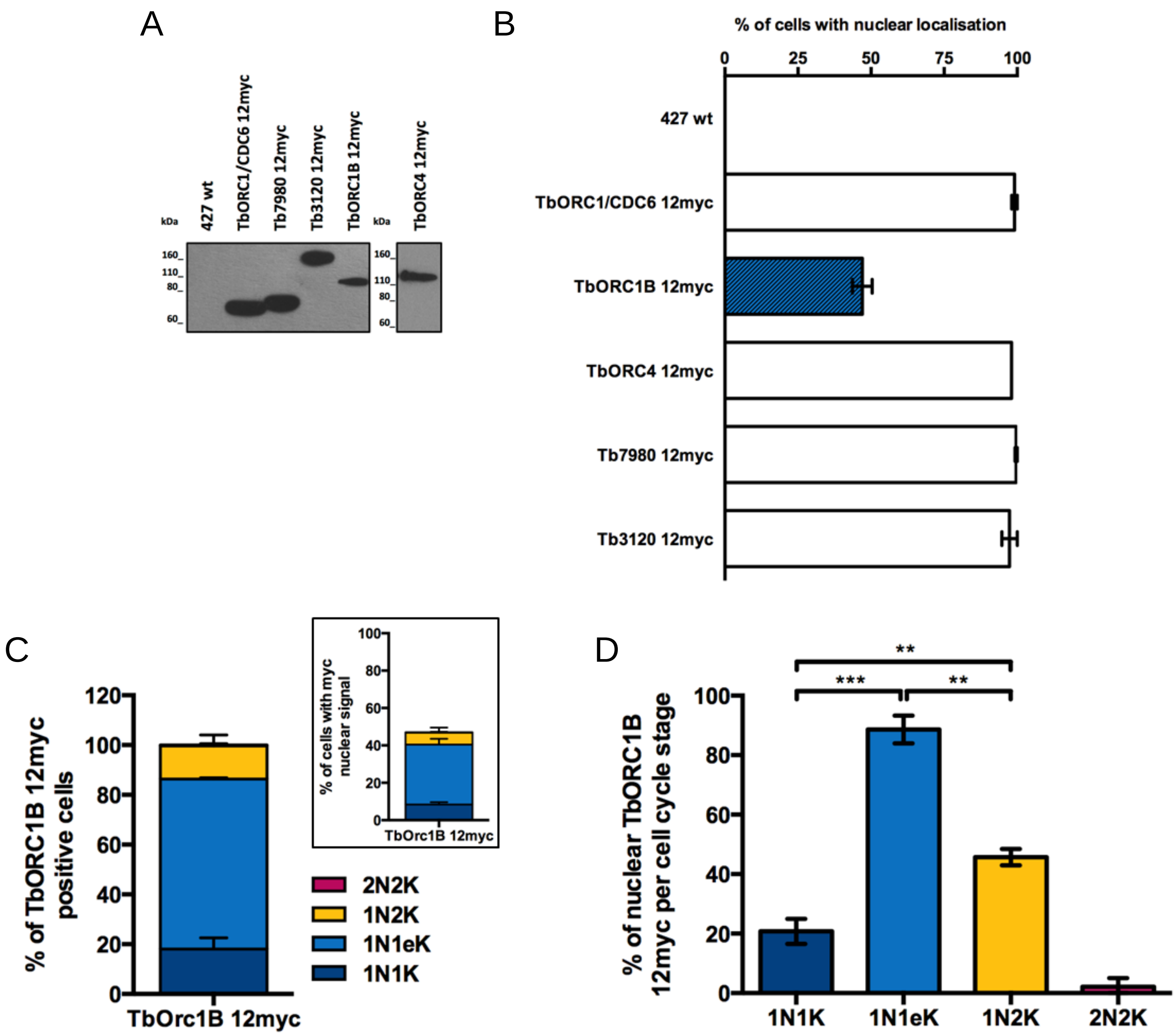
A
B
C
D
kDa
427 wt
TbORC1/CDC6 12myc
Tb7980 12myc
Tb3120 12myc
TbORC1B 12myc
TbORC4 12myc
160
110
80
60
% of cells with nuclear localisation
0
25
50
75
100
427 wt
TbORC1/CDC6 12myc
TbORC1B 12myc
TbORC4 12myc
Tb7980 12myc
Tb3120 12myc
% of TbORC1B 12myc positive cells
120
100
80
60
40
20
0
TbOrc1B 12myc
% of cells with myc nuclear signal
TbOrc1B 12myc
2N2K
1N2K
1N1eK
1N1K
% of nuclear TbORC1B 12myc per cell cycle stage
**
***
**
1N1K
1N1eK
1N2K
2N2K

Fig.S10

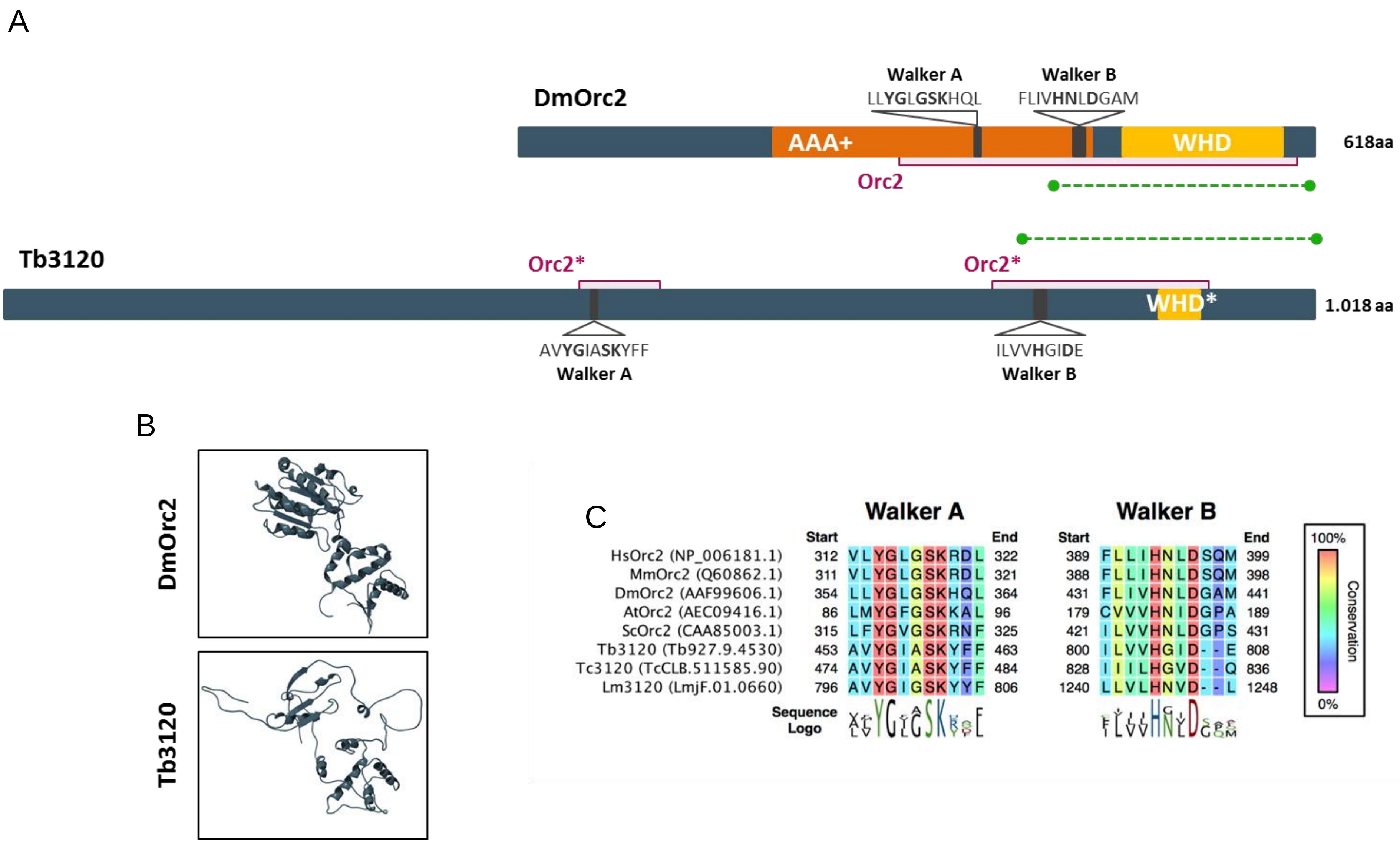
A
DmOrc2
Walker A
LLYGLGSKHQL
Walker B
FLIVHNLDGAM
AAA+
WHD
618aa
Orc2
Tb3120
Orc2*
Orc2*
WHD*
1.018 aa
AVYGIASKYFF
Walker A
ILVVHGIDE
Walker B
B
DmOrc2
Tb3120
C
Walker A
Walker B
Start
End
HsOrc2 (NP_006181.1) 312 VLYGLGSKRDL 322 389 FLLIHNLDSQM 399
MmOrc2 (Q60862.1) 311 VLYGLGSKRDL 321 388 FLLIHNLDSQM 398
DmOrc2 (AAF99606.1) 354 LLYGLGSKHQL 364 431 FLIVHNLDGAM 441
AtOrc2 (AEC09416.1) 86 LMYGFGSKKAL 96 179 CVVVHNIDGPA 189
ScOrc2 (CAA85003.1) 315 LFYGVGSKRNF 325 421 ILVVHNLDGPS 431
Tb3120 (Tb927.9.4530) 453 AVYGIASKYFF 463 800 ILVVHGID--E 808
Tc3120 (TcCLB.511585.90) 474 AVYGIASKYFF 484 828 IIILHGVD--Q 836
Lm3120 (LmjF.01.0660) 796 AVYGIGSKYYF 806 1240 LLVLHNVD--L 1248
Sequence Logo
100%
Conservation
0%

Fig.S11

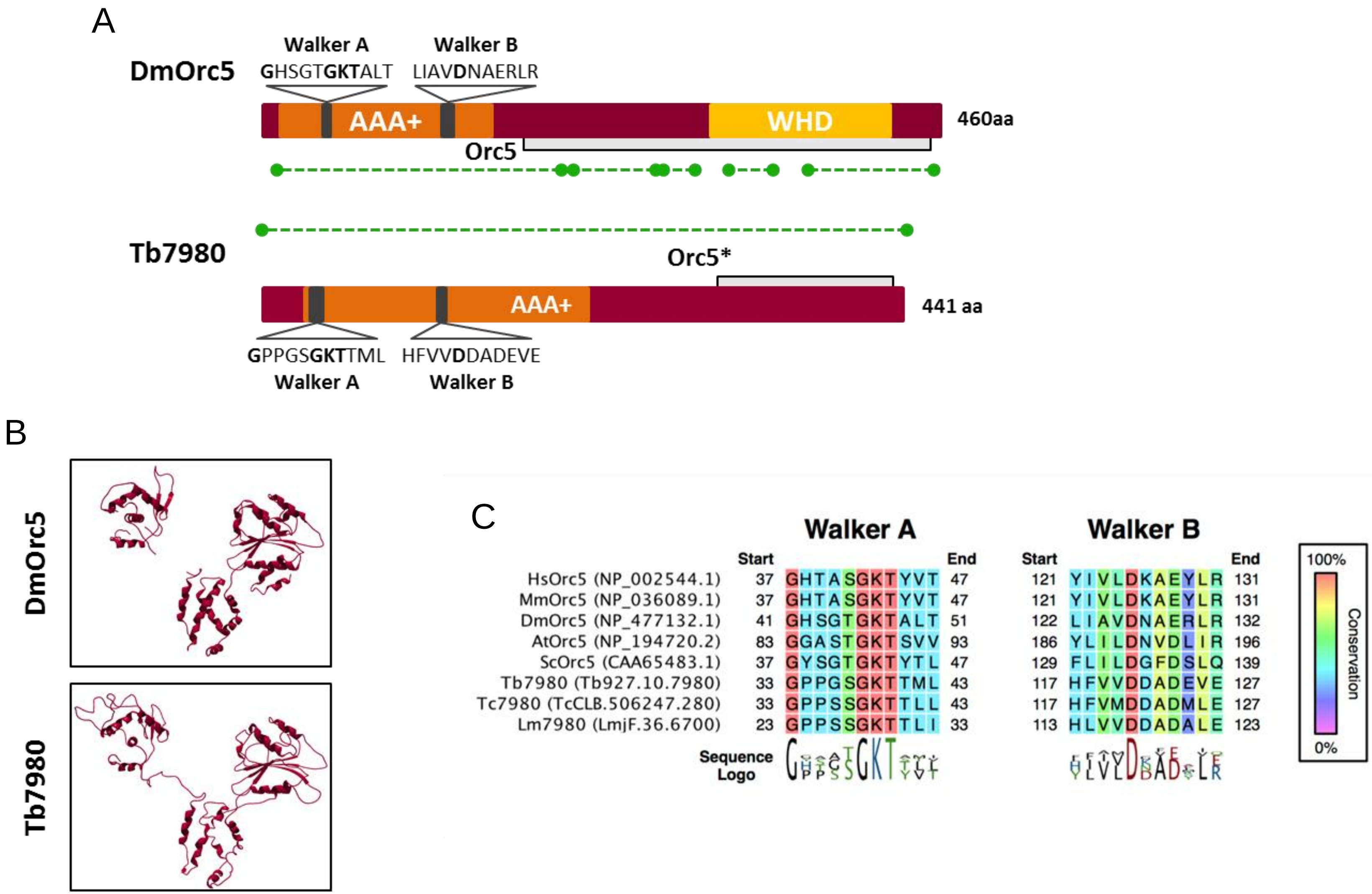
A
DmOrc5
Walker A
GHSGTGKTALT
Walker B
LIAVDNAERLR
AAA+
WHD
460aa
Orc5
Tb7980
Orc5*
AAA+
441 aa
GPPGSGKTTML
Walker A
HFVVDDADEVE
Walker B
B
DmOrc5
Tb7980
C
Walker A
Walker B
Start
End
Start
End
HsOrc5 (NP_002544.1) 37 GHTASGKTYVT 47 121 YIVLDKAEYLR 131
MmOrc5 (NP_036089.1) 37 GHTASGKTYVT 47 121 YIVLDKAEYLR 131
DmOrc5 (NP_477132.1) 41 GHSGTGKTALT 51 122 LIAVDNAERLR 132
AtOrc5 (NP_194720.2) 83 GGASTGKTSVV 93 186 YLILDNVDLIR 196
ScOrc5 (CAA65483.1) 37 GYSGTGKTYTL 47 129 FLILDGFDSLQ 139
Tb7980 (Tb927.10.7980) 33 GPPGSGKTTML 43 117 HFVVDDADEVE 127
Tc7980 (TcCLB.506247.280) 33 GPPSSGKTTLL 43 117 HFVMDDADMLE 127
Lm7980 (LmjF.36.6700) 23 GPPSSGKTTLI 33 113 HLVVDDADALE 123
Sequence Logo
100%
Conservation
0%

Fig.S12
