# Supplementary material for: Diverged composition and regulation of the Trypanosoma brucei origin recognition complex that mediates DNA replication initiation
Source: Nucleic Acids Res. 2016 Mar 6;44(10):4763–84. doi: 10.1093/nar/gkw147 (PMC4889932; doi:10.1093/nar/gkw147)
Supplement: SUPPLEMENTARY DATA [file supp_44_10_4763__index.html]

Diverged composition and regulation of the Trypanosoma brucei origin recognition complex that mediates DNA replication initiation — Diverged composition and regulation of the Trypanosoma brucei origin recognition complex that mediates DNA replication initiation — SUPPLEMENTARY DATA 

# Diverged composition and regulation of the *Trypanosoma brucei* origin recognition complex that mediates DNA replication initiation

## SUPPLEMENTARY DATA

- SUPPLEMENTARY DATA
